# Identification of Significant Secreted or Membrane-Located Proteins in Laryngeal Squamous Cell Carcinoma

**DOI:** 10.1155/2022/9089397

**Published:** 2022-05-23

**Authors:** Li Yan, Chunyan Hu, Yangyang Ji, Lifen Zou, Yang Zhao, Yi Zhu, Xiaoshen Wang

**Affiliations:** ^1^Department of Radiation Oncology, Eye & ENT Hospital, Fudan University, Shanghai, China; ^2^Department of Pathology, Eye & ENT Hospital, Fudan University, Shanghai, China; ^3^Department of Otolaryngology, Eye & ENT Hospital, Fudan University, Shanghai, China

## Abstract

**Background:**

This study is aimed at investigating the expressions and prognostic values of secreted or membrane-located proteins (SMPs) in laryngeal squamous cell carcinoma (LSCC). The correlations between the expressions of SMPs and immune cells' infiltrations were also investigated.

**Methods:**

The expression data of normal laryngeal and LSCC samples were obtained from the TCGA and GEO datasets. The differentially expressed SMPs were identified, and their prognostic values were analyzed. The biological functions of differentially expressed and worse-survival-related SMPs were explored. LASSO regression, Cox multivariate analysis, and nomogram were used to construct a model to predict the survival. Then, the infiltrations of the 24 immune cell populations were calculated using the GSVA method, and the correlations between the expression of SMPs and the immune infiltration were investigated.

**Results:**

122 samples (12 normal and 120 LSCC) of the TCGA database and 114 samples (57 normal and 57 LSCC) of GSE127165 were included. We identified that 138 SMPs were significantly upregulated in LSCC samples of both the TCGA and GEO datasets, among which 52 SMPs were significantly correlated with worse survival. GO and KEGG analyses revealed those 52 SMPs significantly participate in tumor microenvironment and immune cells' communication. Nine of 52 SMPs (ABCC5, ATP1B3, CLEC11A, FLNA, FSTL3, MMP1, NME1, OAS3, and PHLDB2) were included in the nomogram to effectively and accurately predict the LSCC patients' survival. The expressions of most SMPs, such as MMP1 and FSTL3, were significantly positively correlated with the immune infiltration of LSCC.

**Conclusions:**

In this study, the expression, prognostic values, and correlations with immune infiltration of SMPs were analyzed in LSCC samples. Our analyses identified several significant SMPs differentially expressed between normal laryngeal and LSCC samples, correlated with worse survival, and related to the immune infiltration.

## 1. Introduction

Laryngeal cancer is the most common head and neck cancer type, accounting for nearly 185 thousand newly diagnosed cases and 100 thousand death tolls every year [1]. Laryngeal squamous cell carcinoma (LSCC) is the major pathological subtype (about 95%) of laryngeal cancer [2, 3]. Surgery and radiotherapy are the main treatment methods for LSCC. Though the technologies of surgery and radiotherapy have improved a lot, the prognosis of LSCC patients, especially patients at advanced stages, is still not ideal. At present, immunotherapy, such as PD1/PDL1 inhibitors and CAR T therapies, as the most promising cancer therapy, is hopeful of improving the prognosis of LSCC patients significantly [4].

As a major component of the tumor microenvironment, infiltrating immune cells play vital roles in tumor progression, acting as biomarkers as well as targets in immunotherapy [5]. However, infiltrating immune cells are still shrouded in many mysteries, one of which is their dynamic equilibrium interaction with tumor cells. Secreted or membrane-located proteins (SMPs), such as chemokines and PD1/PDL1, as the primary mediators between tumor and immune-infiltrating cells, are of crucial importance in immunotherapy and worthy of further investigation [5, 6].

In this research, we systematically analyzed the expression and prognostic values of SMPs in normal laryngeal and LSCC samples. The correlations between SMPs and infiltrated immune cells were also investigated. We hope our results can help us understand the interaction between infiltrated immune cells and tumor cells and find the biomarkers and potential targets for the immunotherapy of LSCC.

## 2. Methods

### 2.1. Ethics Statement

The study was approved by the Ethics Committee of Eye and ENT Hospital, Fudan University (2020029-1).

### 2.2. Data Source and Processing

Gene expression, clinical, and survival data of Head and Neck Cancer samples of The Cancer Genome Atlas (TCGA) were obtained from the UCSC Xena website (https://gdc.xenahubs.net) [7, 8]. Frozen samples of normal laryngeal tissues and LSCC tissues were included, while recurrent tumor samples (with “-02A” suffix), paraffin-embedded samples (with “-01B” or “-11B” suffix), or samples from other head and neck sites were excluded. Meanwhile, gene expression data of GSE127165 were also included to validate the results obtained from the TCGA data [9]. The fragments per kilobase of exon model per million mapped fragment (FPKM) data of both datasets were log2 (FPKM + 1) transferred before being analyzed.

### 2.3. Identification of Differentially Expressed SMPs

Genes differentially expressed between normal laryngeal and LSCC samples were identified with a threshold of Log2 (fold change) > 0.5 or Log2 (fold change) < −0.5, as well as both *p* value and adjusted *p* value < 0.05 using the limma package of the R software (version 4.0.3) [10]. The list of SMPs was obtained from Human Protein Atlas (https://www.proteinatlas.org/) as previously reported [11, 12].

### 2.4. Survival Analysis

Kaplan-Meier analysis, log-rank test, and Cox multivariate analysis were performed to evaluate the prognostic values of differentially expressed SMPs using the survminer package of the R software. *p* value < 0.05 was considered significantly. Survival curves and the forest plot were plotted using the ggplot2 and the forest plot packages of the R software, respectively.

### 2.5. Gene Ontology (GO) and Kyoto Encyclopedia of Genes and Genomes (KEGG) Analyses

GO and KEGG analyses were performed to investigate the functions of the differentially expressed SMPs with significant prognostic values. The clusterProfiler and the org.Hs.eg.db packages of the R software were used, with a threshold of both adjusted *p* value and false discovery rate (FDR) < 0.05.

### 2.6. Least Absolute Shrinkage and Selection Operator (LASSO) Regression Analysis

LASSO regression was performed in this study to identify the optimal set of differentially expressed SMPs to predict the LSCC patients' survival. The glmnet package of the R software was used, and tenfold cross-validation was used to find the optimal parameter *λ*.

### 2.7. Construction and Validation of the Nomogram

A nomogram was constructed based on the LASSO regression and the Cox multivariate results of survival analyses. Sex, age, and the expression of nine differentially expressed SMPs identified by the LASSO regression were included in the nomogram. The effects of the nomogram were evaluated by the concordance index (C-index). Calibration plots were also calculated using 500 bootstraps. The rms, Hmisc, and ggplot packages of the R software were used as previously reported [13].

### 2.8. Calculation of Immune Cells' Infiltrations

The infiltrations of the 24 immune cell populations were calculated based on the expression profiles of 585 immune cell infiltration-related genes as previously reported using Gene Set Variation Analysis (GSVA) method [13–16]. In this case, GSVA algorithm from the package was a gene enrichment approach that can integrate a specific gene set's relative expression to calculate an enrichment score without supervision. A normalized matrix containing the infiltration enrichment scores ranging from 0 to 1 for each immune cell type in each laryngeal normal or LSCC sample was obtained. The correlations between differentially expressed SMPs and immune cells' infiltrations were calculated and then displayed using heatmaps and scattergraphs. The GSVA, Hmisc, pheatmap, and PerformanceAnalytics packages of the R software were used.

## 3. Results

### 3.1. Identification of Differentially Expressed SMPs

In 613 cases of the TCGA HNSC dataset, 469 cases were excluded due to being not located in the larynx. Then, five cases with suffixes “01B” or “11B” were also excluded since they were paraffin-embedded samples. Seventeen cases were excluded due to the lack of gene expression data. Finally, 122 cases (12 normal laryngeal and 120 LSCC cases) of the TCGA database were included in this research.

**Figure 1:**
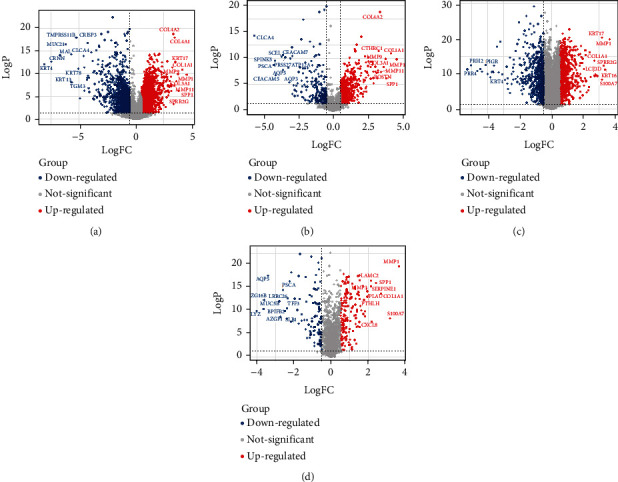
The volcano plots of differentially expressed genes and SMPs: (a) the differentially expressed genes of the TCGA dataset; (b) the differentially expressed SMPs of the TCGA dataset; (c) the differentially expressed genes of GSE127165; (d) the differentially expressed SMPs of GSE127165.

The expression profiles of normal laryngeal and LSCC samples were compared. As Figure 1(a) shows, 3402 genes were significantly upregulated, and 1253 genes were significantly downregulated in LSCC samples. Among them, 669 differentially expressed genes were SMP-coding genes (Figure 1(b)).

Gene expression data of 57 normal laryngeal samples and 57 LSCC samples from GSE127165, the largest LSCC dataset in the GEO database, were also analyzed. As shown in Figures 1(c) and 1(d), 862 genes were significantly upregulated, and 587 genes were significantly downregulated in LSCC samples, among which 247 were SMPs.

After taking the intersection of the results obtained by TCGA and GEO datasets, we found that 138 SMPs were significantly upregulated in LSCC samples of both the TCGA and GEO datasets, including MMP1, COL1A1, SPP1, MMP9, and COL3A1 (Table S1). Meanwhile, 69 SMPs were significantly downregulated in LSCC samples of both the TCGA and GEO datasets, including CLCA4, PSCA, SPINK5, CEACAM5, and SCEL (Table S1).

### 3.2. Identification of Differentially Expressed and Worse-Survival-Related SMPs

**Figure 2:**
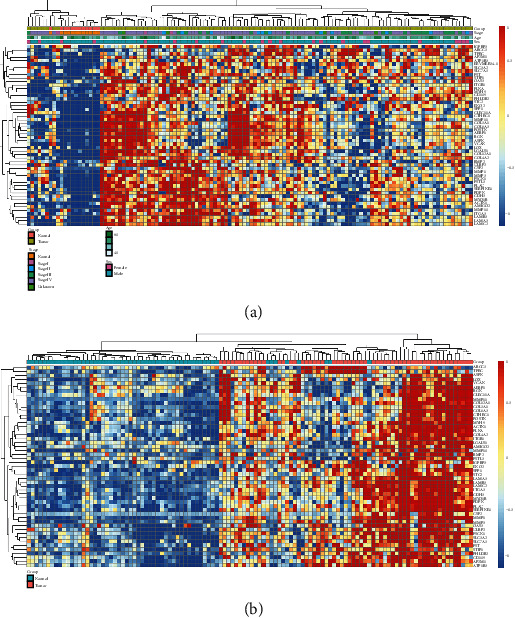
The heatmaps of the differentially expressed and worse-survival-related SMPs: (a) the TCGA dataset; (b) GSE127165.

To further identify the potential immunotherapy targets, we analyzed the correlation between the expression of differentially expressed SMPs and survival, trying to find those proteins associated with worse survival of LSCC patients. Due to the lack of survival data of the GSE127165, only the TCGA samples were analyzed. After calculating the best cut-off values, we found that 52 SMPs were significantly correlated with worse survival, including MMP1, MMP3, LAMA3, STC2, and ATP1B3 (Table S2). The expression profiles of those 52 differentially expressed SMPs in normal laryngeal and LSCC samples were shown in the heatmaps of Figures 2(a) and 2(b), which show that all these SMPs were more highly expressed in LSCC samples than in normal laryngeal samples. Otherwise, 18 differentially expressed SMPs were significantly correlated with better survival, and the correlations between 68 differentially expressed SMPs and survival were not significant (Table S2).

### 3.3. Biological Functions of the Differentially Expressed and Worse-Survival-Related SMPs

**Figure 3:**
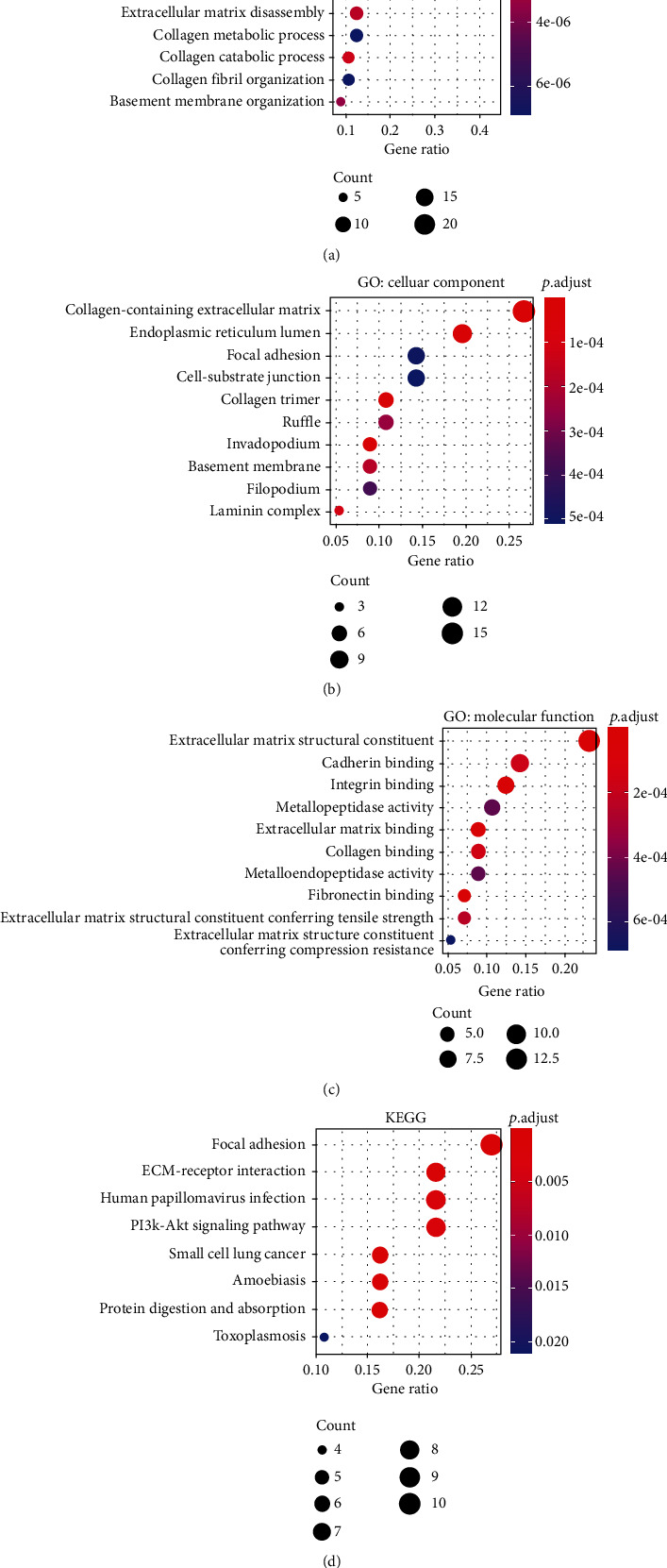
The GO and KEGG analyses' results of the differentially expressed and worse-survival-related SMPs: (a) GO: biological process; (b) GO: cellular component; (c) GO: molecular functions; (d) KEGG.

We investigated in which GOs and signaling pathways the SMPs both differentially expressed and correlated with worse survival were enriched, to explore those SMPs' biological function. As Figures 3(a)–3(c) show, those 52 SMPs were significantly enriched in the GOs such as extracellular matrix organization, collagen catabolic process, cadherin binding, and integrin binding. Most enriched GOs were related to tumor microenvironment or immune cells' communication, indicating that the SMPs may play their functions in immune cells' infiltration. Meanwhile, those SMPs were also enriched in the pathways such as focal adhesion, ECM-receptor interaction, and PI3K/Akt pathway (Figure 3(d)), which also suggested that those SMPs significantly participated in the tumor microenvironment.

### 3.4. Constructions of the Prognosis Prediction Model

**Figure 4:**
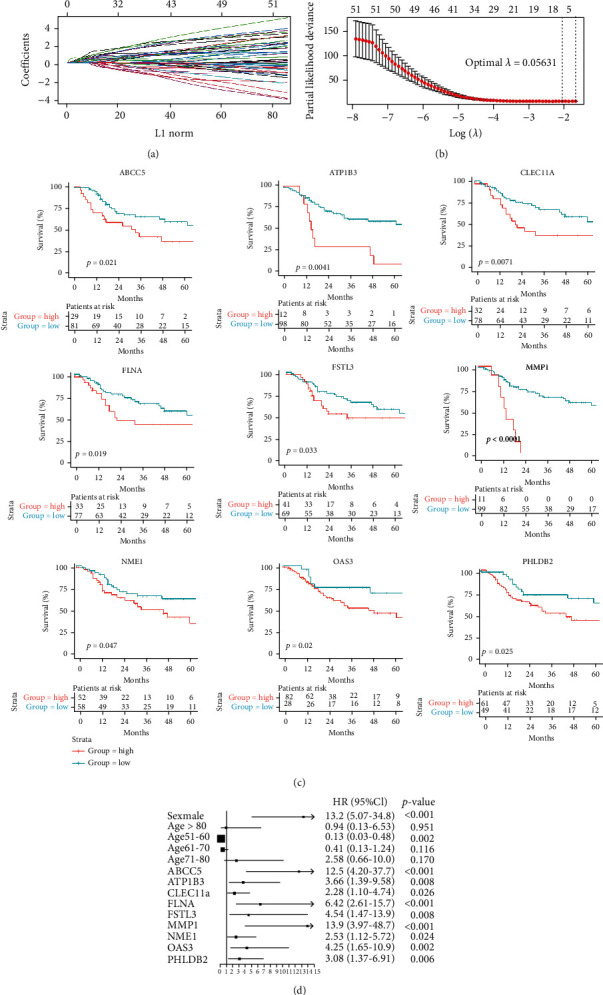
SMPs in the LASSO regression model to predict the survival of LSCC: (a) coefficient profiles of variables in the LASSO regression model; (b) tenfold cross-validation for selecting parameter *λ* in the LASSO regression model; (c) survival curves of the selected SMPs; (d) forest plot of the Cox multivariate analysis.

LASSO regression method uses a penalty function to limit the number of factors included and get a more refined regression model, which is now widely used in genetic prediction [17, 18]. In this research, we used LASSO regression to select the optimal SMPs for constructing the prognosis prediction model. Finally, nine of 52 SMPs (ABCC5, ATP1B3, CLEC11A, FLNA, FSTL3, MMP1, NME1, OAS3, and PHLDB2) were selected in the model when lambda.min was chosen as the optimal parameter *λ* in the LASSO analysis (Figures 4(a) and 4(b)). The survival curves of the nine SMPs are displayed in Figure 4(c), showing that those genes were significantly correlated with worse survival of LSCC patients.

As Figure 4(d) shows, the multivariate Cox analysis demonstrated that the expressions of the nine SMPs were independent prognostic factors, as well as age and sex. However, tumor stage was not identified as an independent prognostic factor with a 0.674 *p* value.

**Figure 5:**
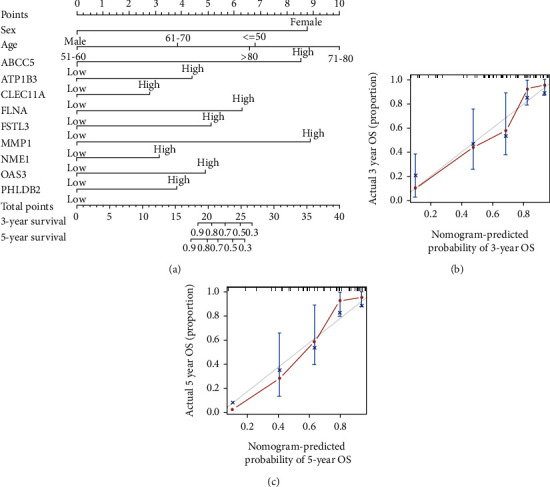
Construction and validation of the nomogram: (a) the nomogram using the expressions of SMPs to predict LSCC patients' overall survival; (b) calibration curve of the nomogram predicting 3-year overall survival rate; (c) calibration curve of the nomogram predicting 5-year overall survival.

Based on the results of the multivariate Cox analysis, we constructed a nomogram to visually predict the LSCC patients' survival (Figure 5(a)). The C-index (0.877 ± 0.047) and the calibration curves (Figures 5(b) and 5(c)) showed that the nomogram could effectively and accurately predict survival.

### 3.5. Correlations between the SMPs and Infiltration of Immune Cells

To investigate the functions of differentially expressed and worse-survival-related SMPs in immune infiltration of LSCC samples, we analyzed the correlations between those SMPs and the infiltrations of immune cells. The scores of 24 immune cell populations, including most types of T cells, NK cells, and macrophages, were calculated based on the GSVA method.

**Figure 6:**
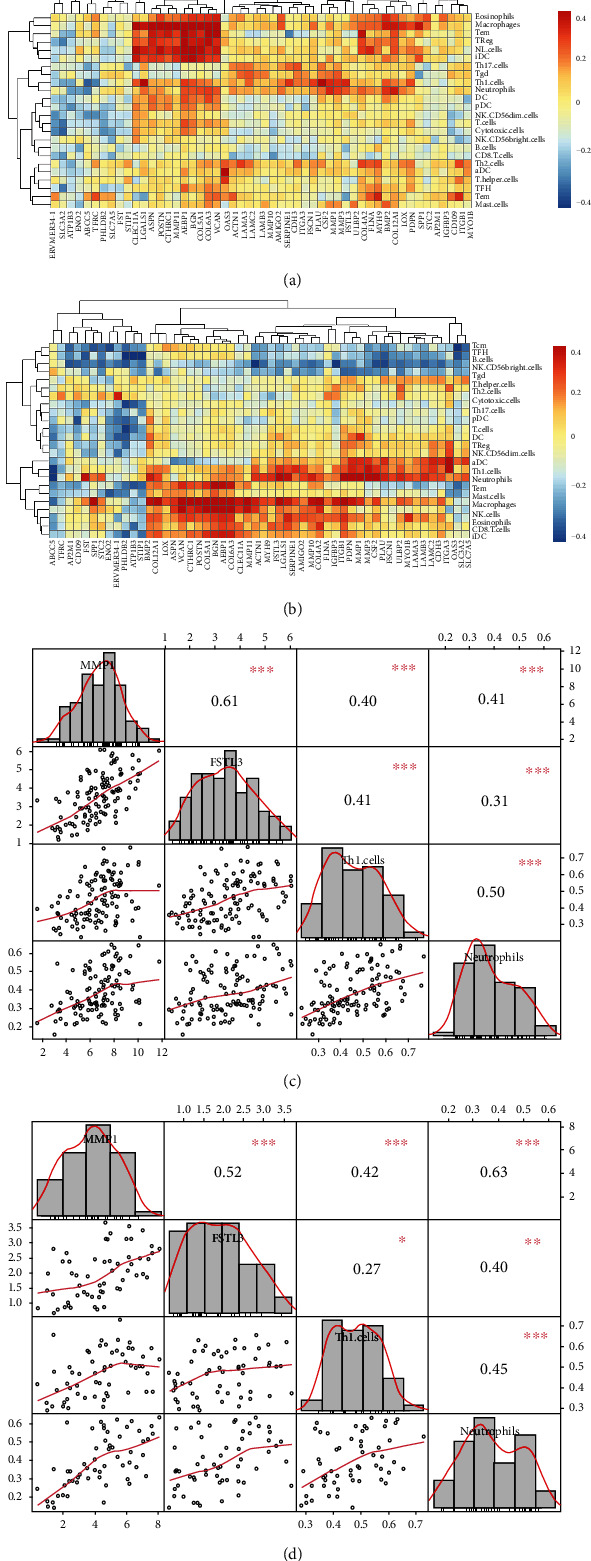
Correlations between differentially expressed, worse-survival-related SMPs, and the infiltrations of immune cells: (a) correlations in the TCGA dataset; (b) correlations in GSE127165; (c) correlations among MMP1, FSTL3, Th1 cells, and neutrophils in the TCGA dataset; (d) correlations among MMP1, FSTL3, Th1 cells, and neutrophils in GSE127165.

As Figures 6(a) and 6(b) show, the expressions of most SMPs, such as MMP1, FSTL3, OAS3, FLNA, and CLEC11A, were significantly positively correlated with the immune infiltration in LSCC samples of both the TCGA and the GEO datasets, indicating those SMPs were related to the higher immune infiltration of LSCC. For example, the expressions of MMP1 and FSTL3 were significantly positively correlated with the infiltration scores of Th1 cells and neutrophils (Figure 6(c)). Meanwhile, the expressions of a small part of SMPs were negatively correlated with the immune infiltration, such as NME1 and ATP1B3.

The infiltrations of Th1 cells, neutrophils, macrophages, eosinophils, and immature dendritic cells (iDC) were more positively correlated with the expressions of SMPs, while B cells and NK CD56dim cells were more negatively correlated with the expressions of SMPs (Figures 6(a) and 6(b)). Those results suggest the different roles of SMPs in the infiltrations of different immune cells.

## 4. Discussion

In this study, we investigated the differentially expressed SMPs in LSCC samples and their prognostic values. We found that differentially expressed and worse-survival-related SMPs mainly participated in GOs and pathways related to immune cells' infiltrations, and the expressions of many SMPs significantly correlated with the scores of immune cells' infiltrations. Our results identified some significant SMPs which might play important roles in the immune cells' infiltrations and have the potential to the biomarkers and potential targets for the immunotherapy of LSCC.

We took a deep look into the SMPs of LSCC in this research, since SMPs are the primary mediators in the interaction between tumor and immune-infiltrating cells. Both tumor and immune-infiltrating cells secrete a lot of chemokines, cytokines, and growth factors, inducing the migration, colonization, and differentiation of immune cells as well as the proliferation and progression of tumor cells [5, 12]. Meanwhile, tumor and immune-infiltrating cells also directly interact with each other via the ligands and receptors located in their membranes [5, 12]. Therefore, studying SMPs can help us better understand carcinogenesis, and SMPs have great potential in cancer immunotherapy. Targeting SMPs such as PD1/PD-L1 and CTLA4 has been commonly used in the clinic, including in the treatment of LSCC [19–22]. Using CAR T cells with cancer-specific SMPs or coupling SMPs' antibodies with chemotherapeutic agents is also developing rapidly [23–25].

Our results showed that MMP1 and FSTL3 were significantly upregulated in LSCC and positively correlated with worse survival as well as several immune cells' infiltrations. MMP1 greatly promotes tumor cells' migration and invasion and has been reported to correlate with immune cells' infiltrations in breast and cervical cancer [26, 27]. Eiro et al. [28] found that MMP1 mediated the interaction among peripheral blood mononuclear cells, cancer cells, and cancer-associated fibroblasts. Yang et al. [29] reported that FSTL3 promoted macrophage and fibroblast polarization and T cell exhaustion, forming an inhibitory immune microenvironment and accelerating metastasis in colorectal cancer. They found that high FSTL3 expression is linked to immunotherapy-sensitive [29].

Numerous studies have reported the important roles of immune cells' infiltrations in the carcinogenesis and treatment of LSCC and other tumors [14, 30, 31]. However, it is not easy to investigate the infiltrated immune cells in tumor samples. Nowadays, with the rapidly developing high-throughput single-cell sequencing technology, each immune cell's cellular and molecular characteristics can be more easily explored. Though there is still a lack of single-cell sequencing data for LSCC, we hope that more data in the future can better study the LSCC's immune infiltration.

There are several limitations of our study. First, there is no survival data of the GEO data included in our study. Therefore, we can not analyze whether the expressions of our identified SMPs were still correlated with survival in the GEO dataset. Second, the validation cohort only included 57 LSCC patients in the GEO database. If the sample size in the validation cohort was larger, the results would be more convinced. Third, because of the lack of an LSCC cohort with immunotherapy, we could not analyze the expressions and functions of our identified SMPs in immunotherapy-treated LSCC patients, which is also a big limitation of our study.

## 5. Conclusions

In this study, the expression, prognostic values, and correlations with immune infiltration of SMPs were analyzed in LSCC samples. Our analyses identified several significant SMPs differentially expressed between normal laryngeal and LSCC samples, correlated with worse survival, and related to the immune infiltration. We hope our results can help us understand the interaction between infiltrated immune cells and tumor cells and find the biomarkers and potential targets for the immunotherapy of LSCC.

## Data Availability

Data can be obtained from the UCSC Xena browser (https://gdc.xenahubs.net) and the GEO database (https://www.ncbi.nlm.nih.gov/gds/) under the accession numbers GSE127165.

## Supplementary Materials

Supplementary 1 Table S1: genes differentially expressed in the TCGA and the GEO databases.

Supplementary 2 Table S2: survival analyses of differentially expressed SMPs.
