# Supplementary material for: Identification of Significant Secreted or Membrane-Located Proteins in Laryngeal Squamous Cell Carcinoma
Source: J Immunol Res. 2022 May 23;2022:9089397. doi: 10.1155/2022/9089397 (PMC9153386; doi:10.1155/2022/9089397)
Supplement: Supplementary 1 — Table S1: genes differentially expressed in the TCGA and the GEO databases. [file 9089397.f1.docx]

Table S1. Genes differentially expressed in the TCGA and the GEO databases

| Gene.name | membrene protein | secreted protein | logFC_TCGA | AveExpr_TCGA | t_TCGA | P.Value_TCGA | adj.P.Val_TCGA | B_TCGA | logP_TCGA | Group_TCGA | logFC_GEO | AveExpr_GEO | t_GEO | P.Value_GEO | adj.P.Val_GEO | B_GEO | logP_GEO | Group_GEO |
| --- | --- | --- | --- | --- | --- | --- | --- | --- | --- | --- | --- | --- | --- | --- | --- | --- | --- | --- |
| MMP1 | No | Yes | 4.566907 | 6.168848 | 7.908368 | 1.39E-12 | 1.47E-10 | 18.23055 | 9.832348 | up-regulated | 3.659044 | 2.531322 | 12.60994 | 2.80E-23 | 1.55E-20 | 42.40331 | 19.81056 | up-regulated |
| COL1A1 | No | Yes | 4.174104 | 7.661132 | 8.44696 | 7.83E-14 | 1.25E-11 | 21.03319 | 10.90136 | up-regulated | 2.700592 | 5.142116 | 9.498141 | 4.49E-16 | 1.75E-14 | 26.04835 | 13.75606 | up-regulated |
| CLCA4 | Yes | No | -5.68708 | 2.699035 | -10.0942 | 9.56E-18 | 5.24E-15 | 29.82546 | 14.28098 | down-regulated | -2.35566 | 2.172405 | -9.02477 | 5.57E-15 | 1.68E-13 | 23.56815 | 12.77493 | down-regulated |
| PSCA | Yes | No | -4.29534 | 2.963921 | -7.34733 | 2.61E-11 | 1.81E-09 | 15.37275 | 8.741851 | down-regulated | -2.59967 | 3.063212 | -9.92 | 4.72E-17 | 2.50E-15 | 28.26937 | 14.60131 | down-regulated |
| SPP1 | No | Yes | 3.838767 | 4.971833 | 5.954632 | 2.63E-08 | 6.34E-07 | 8.665971 | 6.19762 | up-regulated | 2.415729 | 2.196937 | 10.66961 | 8.53E-19 | 8.04E-17 | 32.22631 | 16.0947 | up-regulated |
| MMP9 | No | Yes | 3.774705 | 4.708627 | 7.9517 | 1.10E-12 | 1.22E-10 | 18.45409 | 9.912857 | up-regulated | 1.781271 | 1.993072 | 8.83214 | 1.55E-14 | 4.12E-13 | 22.56404 | 12.38513 | up-regulated |
| COL3A1 | No | Yes | 3.632395 | 7.549902 | 7.121335 | 8.35E-11 | 4.77E-09 | 14.24319 | 8.321522 | up-regulated | 1.8318 | 4.629801 | 7.094265 | 1.22E-10 | 1.29E-09 | 13.74748 | 8.889897 | up-regulated |
| SPINK5 | No | Yes | -4.20352 | 4.775431 | -7.77293 | 2.84E-12 | 2.80E-10 | 17.53427 | 9.553419 | down-regulated | -1.15544 | 1.862946 | -4.48567 | 1.76E-05 | 6.14E-05 | 2.205581 | 4.211872 | down-regulated |
| CEACAM5 | Yes | No | -4.15803 | 4.4605 | -6.30366 | 4.93E-09 | 1.55E-07 | 10.28414 | 6.809419 | down-regulated | -1.96893 | 3.099542 | -8.9689 | 7.49E-15 | 2.19E-13 | 23.27653 | 12.66004 | down-regulated |
| MMP11 | No | Yes | 3.437686 | 3.5649 | 6.614348 | 1.07E-09 | 4.16E-08 | 11.76269 | 7.380403 | up-regulated | 1.472547 | 0.948698 | 9.734828 | 1.27E-16 | 5.89E-15 | 27.29354 | 14.23015 | up-regulated |
| COL4A2 | No | Yes | 3.373469 | 5.472174 | 12.30396 | 4.73E-23 | 1.51E-19 | 41.73955 | 18.82224 | up-regulated | 0.940972 | 1.11702 | 11.38344 | 1.87E-20 | 3.17E-18 | 35.99009 | 17.49898 | up-regulated |
| SCEL | Yes | No | -3.73622 | 3.436214 | -8.13826 | 4.09E-13 | 5.21E-11 | 19.42061 | 10.28348 | down-regulated | -1.09442 | 1.419512 | -5.17575 | 9.99E-07 | 4.56E-06 | 4.966022 | 5.340803 | down-regulated |
| CEACAM7 | Yes | No | -3.64134 | 1.725492 | -8.28703 | 1.85E-13 | 2.66E-11 | 20.1958 | 10.57521 | down-regulated | -0.68829 | 1.044368 | -3.57532 | 0.000516 | 0.001309 | -0.99904 | 2.883086 | down-regulated |
| CTHRC1 | No | Yes | 3.285233 | 4.051268 | 8.675045 | 2.29E-14 | 4.25E-12 | 22.23404 | 11.37147 | up-regulated | 1.29962 | 1.309539 | 10.05964 | 2.24E-17 | 1.33E-15 | 29.00587 | 14.8755 | up-regulated |
| AQP5 | Yes | No | -3.62688 | 1.978673 | -6.83733 | 3.52E-10 | 1.61E-08 | 12.84397 | 7.793807 | down-regulated | -3.42157 | 2.965441 | -11.4883 | 1.07E-20 | 2.01E-18 | 36.54167 | 17.69662 | down-regulated |
| POSTN | No | Yes | 3.227819 | 4.724742 | 6.595421 | 1.18E-09 | 4.51E-08 | 11.67166 | 7.345595 | up-regulated | 1.819073 | 2.182993 | 7.647822 | 7.40E-12 | 1.04E-10 | 16.49525 | 9.981743 | up-regulated |
| PRSS27 | No | Yes | -3.46664 | 2.294077 | -8.05721 | 6.30E-13 | 7.44E-11 | 18.99989 | 10.12839 | down-regulated | -1.29969 | 2.138766 | -4.4324 | 2.17E-05 | 7.43E-05 | 2.00361 | 4.128989 | down-regulated |
| ATP12A | Yes | No | -3.24893 | 2.051262 | -7.04845 | 1.21E-10 | 6.45E-09 | 13.88186 | 8.190475 | down-regulated | -1.29154 | 1.449577 | -6.65831 | 1.05E-09 | 9.00E-09 | 11.63872 | 8.045631 | down-regulated |
| LAMC2 | No | Yes | 3.225829 | 6.844169 | 6.792302 | 4.42E-10 | 1.96E-08 | 12.62432 | 7.708125 | up-regulated | 2.147117 | 2.241302 | 10.94776 | 1.92E-19 | 2.17E-17 | 33.69434 | 16.66258 | up-regulated |
| MMP3 | No | Yes | 3.16965 | 3.687435 | 5.936011 | 2.87E-08 | 6.78E-07 | 8.581007 | 6.168809 | up-regulated | 1.873177 | 1.459692 | 10.02047 | 2.76E-17 | 1.59E-15 | 28.79925 | 14.79937 | up-regulated |
| AQP3 | Yes | No | -3.18049 | 6.832992 | -5.48937 | 2.26E-07 | 3.85E-06 | 6.588144 | 5.414536 | down-regulated | -1.82339 | 5.488013 | -7.79698 | 3.44E-12 | 5.30E-11 | 17.24702 | 10.27604 | down-regulated |
| COL1A2 | No | Yes | 3.129196 | 6.775663 | 6.513861 | 1.76E-09 | 6.41E-08 | 11.28078 | 7.193192 | up-regulated | 1.510568 | 4.355715 | 6.429649 | 3.18E-09 | 2.47E-08 | 10.55547 | 7.606557 | up-regulated |
| COL6A3 | No | Yes | 3.044659 | 4.425841 | 7.182414 | 6.11E-11 | 3.64E-09 | 14.54713 | 8.43929 | up-regulated | 1.398605 | 2.58179 | 7.822401 | 3.02E-12 | 4.71E-11 | 17.37557 | 10.32678 | up-regulated |
| PTHLH | No | Yes | 3.024591 | 5.140528 | 6.646423 | 9.15E-10 | 3.61E-08 | 11.91723 | 7.442686 | up-regulated | 1.954933 | 1.64914 | 9.191071 | 2.31E-15 | 7.63E-14 | 24.43761 | 13.11743 | up-regulated |
| CA9 | Yes | No | 2.906717 | 3.047978 | 5.372465 | 3.83E-07 | 5.93E-06 | 6.081638 | 5.227025 | up-regulated | 0.658946 | 0.407515 | 5.820612 | 5.60E-08 | 3.33E-07 | 7.759671 | 6.477364 | up-regulated |
| COL5A1 | No | Yes | 2.897859 | 4.474293 | 6.654141 | 8.81E-10 | 3.49E-08 | 11.95447 | 7.457611 | up-regulated | 0.87071 | 1.00136 | 6.590824 | 1.46E-09 | 1.22E-08 | 11.31726 | 7.914005 | up-regulated |
| FAM3B | No | Yes | -3.09322 | 1.703315 | -7.96723 | 1.02E-12 | 1.14E-10 | 18.5343 | 9.941223 | down-regulated | -0.65266 | 0.531007 | -12.2474 | 1.90E-22 | 7.42E-20 | 40.5176 | 19.12939 | down-regulated |
| SERPINE1 | No | Yes | 2.884616 | 5.79756 | 5.983734 | 2.29E-08 | 5.68E-07 | 8.79904 | 6.245438 | up-regulated | 2.156278 | 2.176393 | 10.32345 | 5.44E-18 | 3.90E-16 | 30.39846 | 15.40899 | up-regulated |
| SPARC | No | Yes | 2.834609 | 7.308505 | 7.639987 | 5.70E-12 | 4.99E-10 | 16.85463 | 9.301489 | up-regulated | 1.317485 | 3.780309 | 6.853819 | 4.02E-10 | 3.79E-09 | 12.57772 | 8.421933 | up-regulated |
| BGN | No | Yes | 2.814183 | 6.793957 | 7.385936 | 2.14E-11 | 1.51E-09 | 15.56705 | 8.821987 | up-regulated | 0.811553 | 2.842271 | 4.265646 | 4.16E-05 | 0.000133 | 1.382333 | 3.874616 | up-regulated |
| FST | No | Yes | 2.710549 | 4.408793 | 7.668432 | 4.91E-12 | 4.49E-10 | 16.99973 | 9.347435 | up-regulated | 1.49797 | 2.341085 | 9.342983 | 1.03E-15 | 3.65E-14 | 25.2337 | 13.43828 | up-regulated |
| PPL | Yes | No | -2.96817 | 5.853165 | -9.01023 | 3.70E-15 | 8.48E-13 | 24.01095 | 12.07166 | down-regulated | -1.12257 | 3.029613 | -4.84642 | 4.05E-06 | 1.63E-05 | 3.616509 | 4.788746 | down-regulated |
| APOE | No | Yes | 2.709102 | 4.902536 | 5.887883 | 3.60E-08 | 8.28E-07 | 8.362084 | 6.081709 | up-regulated | 0.99954 | 3.955993 | 5.612227 | 1.45E-07 | 7.85E-07 | 6.836583 | 6.105249 | up-regulated |
| COL6A1 | No | Yes | 2.705148 | 6.197052 | 7.127255 | 8.10E-11 | 4.65E-09 | 14.2726 | 8.332137 | up-regulated | 0.84602 | 3.511428 | 4.714772 | 6.97E-06 | 2.66E-05 | 3.093137 | 4.575666 | up-regulated |
| PLAU | No | Yes | 2.513779 | 5.980016 | 7.22871 | 4.82E-11 | 2.94E-09 | 14.77818 | 8.531849 | up-regulated | 1.960126 | 2.862306 | 9.775785 | 1.02E-16 | 4.87E-15 | 27.50927 | 14.31215 | up-regulated |
| THY1 | Yes | No | 2.500793 | 3.454649 | 8.078347 | 5.63E-13 | 6.73E-11 | 19.10951 | 10.17181 | up-regulated | 1.313589 | 2.017309 | 8.978316 | 7.13E-15 | 2.09E-13 | 23.32567 | 12.67969 | up-regulated |
| FUT6 | No | Yes | -2.96572 | 1.562633 | -8.20541 | 2.86E-13 | 3.89E-11 | 19.77005 | 10.41019 | down-regulated | -1.67705 | 1.544673 | -13.9031 | 3.33E-26 | 4.16E-23 | 49.03849 | 22.38075 | down-regulated |
| ALDH3A1 | Yes | No | -2.95365 | 5.488371 | -3.99945 | 0.00011 | 0.000705 | 0.687811 | 3.151593 | down-regulated | -1.74272 | 3.613267 | -6.25917 | 7.19E-09 | 5.14E-08 | 9.759198 | 7.289199 | down-regulated |
| COL12A1 | No | Yes | 2.408363 | 4.18729 | 6.596528 | 1.17E-09 | 4.50E-08 | 11.67698 | 7.347133 | up-regulated | 1.447631 | 1.905974 | 11.48359 | 1.10E-20 | 2.04E-18 | 36.51686 | 17.69001 | up-regulated |
| TMPRSS11E | No | Yes | -2.85305 | 3.848329 | -5.12107 | 1.16E-06 | 1.52E-05 | 5.01526 | 4.818383 | down-regulated | -0.50786 | 1.275815 | -2.54992 | 0.012116 | 0.02256 | -3.89551 | 1.646668 | down-regulated |
| ADH7 | Yes | No | -2.67507 | 3.928098 | -4.37406 | 2.60E-05 | 0.000207 | 2.048717 | 3.684369 | down-regulated | -1.13952 | 2.22728 | -5.36023 | 4.46E-07 | 2.19E-06 | 5.745777 | 5.659906 | down-regulated |
| COL10A1 | No | Yes | 2.376369 | 2.249171 | 4.963052 | 2.30E-06 | 2.68E-05 | 4.361642 | 4.571622 | up-regulated | 0.581563 | 0.656114 | 6.646294 | 1.11E-09 | 9.50E-09 | 11.58136 | 8.022136 | up-regulated |
| ZG16B | No | Yes | -2.67425 | 1.875828 | -5.0875 | 1.35E-06 | 1.71E-05 | 4.87529 | 4.767199 | down-regulated | -3.61843 | 2.986853 | -8.82462 | 1.61E-14 | 4.26E-13 | 22.52493 | 12.37096 | down-regulated |
| ANXA1 | Yes | Yes | -2.4898 | 8.215621 | -6.62853 | 1.00E-09 | 3.90E-08 | 11.831 | 7.408408 | down-regulated | -0.90081 | 6.175328 | -3.15461 | 0.00206 | 0.004572 | -2.28623 | 2.339934 | down-regulated |
| SERPINB1 | No | Yes | -2.40388 | 6.268421 | -7.09752 | 9.43E-11 | 5.29E-09 | 14.12497 | 8.276198 | down-regulated | -1.09803 | 4.695047 | -4.89267 | 3.33E-06 | 1.36E-05 | 3.802588 | 4.865828 | down-regulated |
| IL36G | Yes | Yes | 2.373136 | 4.065351 | 4.305177 | 3.41E-05 | 0.000261 | 1.791745 | 3.583508 | up-regulated | 1.328612 | 1.08098 | 7.234671 | 6.03E-11 | 6.85E-10 | 14.4376 | 9.164482 | up-regulated |
| ENO2 | Yes | No | 2.363658 | 3.257594 | 7.594329 | 7.24E-12 | 6.05E-10 | 16.62211 | 9.218504 | up-regulated | 0.665783 | 1.391596 | 7.086477 | 1.27E-10 | 1.33E-09 | 13.70935 | 8.874706 | up-regulated |
| MUC1 | Yes | Yes | -2.27408 | 3.864651 | -5.67371 | 9.75E-08 | 1.90E-06 | 7.399885 | 5.722271 | down-regulated | -1.74647 | 4.087632 | -11.4593 | 1.25E-20 | 2.21E-18 | 36.38916 | 17.65475 | down-regulated |
| AEBP1 | No | Yes | 2.361961 | 5.317878 | 6.004308 | 2.08E-08 | 5.24E-07 | 8.893328 | 6.280627 | up-regulated | 0.583911 | 3.908124 | 3.261106 | 0.001468 | 0.003368 | -1.97289 | 2.472633 | up-regulated |
| ODC1 | Yes | No | 2.328844 | 6.565248 | 4.744306 | 5.79E-06 | 5.86E-05 | 3.479272 | 4.232236 | up-regulated | 1.632831 | 3.81305 | 7.74893 | 4.41E-12 | 6.61E-11 | 17.00437 | 10.18 | up-regulated |
| LGALS1 | No | Yes | 2.314854 | 7.506295 | 6.725296 | 6.18E-10 | 2.60E-08 | 12.29869 | 7.585588 | up-regulated | 0.801119 | 3.783308 | 4.144988 | 6.60E-05 | 0.000203 | 0.94352 | 3.692897 | up-regulated |
| COL7A1 | No | Yes | 2.296982 | 4.788363 | 8.175517 | 3.36E-13 | 4.47E-11 | 19.61438 | 10.34974 | up-regulated | 1.532678 | 3.217183 | 10.98355 | 1.59E-19 | 1.87E-17 | 33.88313 | 16.72702 | up-regulated |
| THBS2 | Yes | Yes | 2.293867 | 4.154121 | 5.717113 | 7.98E-08 | 1.60E-06 | 7.593262 | 5.795016 | up-regulated | 1.253653 | 1.856787 | 7.146275 | 9.39E-11 | 1.02E-09 | 14.00254 | 8.993149 | up-regulated |
| KLHDC7B | Yes | No | 2.224185 | 2.347392 | 4.335473 | 3.03E-05 | 0.000235 | 1.904405 | 3.628162 | up-regulated | 1.428561 | 1.623547 | 5.950924 | 3.07E-08 | 1.92E-07 | 8.346044 | 6.715863 | up-regulated |
| KRT75 | Yes | No | 2.149361 | 2.831303 | 3.62425 | 0.000425 | 0.002189 | -0.58106 | 2.659781 | up-regulated | 1.387545 | 0.936979 | 7.616745 | 8.68E-12 | 1.20E-10 | 16.33918 | 9.919179 | up-regulated |
| LRRC15 | Yes | No | 2.048274 | 2.047053 | 4.728964 | 6.17E-06 | 6.19E-05 | 3.418394 | 4.208076 | up-regulated | 1.115579 | 0.795406 | 7.998536 | 1.22E-12 | 2.09E-11 | 18.26949 | 10.68064 | up-regulated |
| HOMER3 | Yes | No | 2.020579 | 3.897915 | 10.01766 | 1.46E-17 | 7.38E-15 | 29.41271 | 14.13207 | up-regulated | 0.910593 | 1.396736 | 10.79907 | 4.26E-19 | 4.44E-17 | 32.90974 | 16.35215 | up-regulated |
| ASPN | No | Yes | 2.015648 | 2.789137 | 4.680703 | 7.54E-06 | 7.31E-05 | 3.227774 | 4.136082 | up-regulated | 0.821862 | 1.10154 | 5.86818 | 4.50E-08 | 2.73E-07 | 7.972923 | 6.56379 | up-regulated |
| CXCL8 | No | Yes | 2.009863 | 4.500675 | 4.037574 | 9.52E-05 | 0.000624 | 0.822136 | 3.204996 | up-regulated | 2.180662 | 4.109504 | 6.491752 | 2.36E-09 | 1.89E-08 | 10.84799 | 7.723626 | up-regulated |
| STC2 | No | Yes | 2.009626 | 2.232448 | 6.502661 | 1.86E-09 | 6.68E-08 | 11.22728 | 7.175161 | up-regulated | 0.738033 | 0.703459 | 9.510749 | 4.20E-16 | 1.66E-14 | 26.11461 | 13.77991 | up-regulated |
| TMPRSS2 | Yes | Yes | -2.18685 | 1.61919 | -6.48439 | 2.04E-09 | 7.26E-08 | 11.1401 | 7.139263 | down-regulated | -0.67906 | 0.592231 | -13.0101 | 3.43E-24 | 2.68E-21 | 44.47294 | 20.5715 | down-regulated |
| PRAME | Yes | No | 2.002102 | 1.843379 | 4.144239 | 6.36E-05 | 0.000442 | 1.203057 | 3.354561 | up-regulated | 0.760087 | 0.471347 | 6.606085 | 1.36E-09 | 1.14E-08 | 11.38982 | 7.943526 | up-regulated |
| LAMA3 | No | Yes | 1.947986 | 4.36013 | 4.841572 | 3.85E-06 | 4.20E-05 | 3.868333 | 4.377203 | up-regulated | 0.870849 | 0.917892 | 9.53643 | 3.66E-16 | 1.47E-14 | 26.2496 | 13.83194 | up-regulated |
| FSTL3 | No | Yes | 1.858226 | 3.363708 | 5.172787 | 9.28E-07 | 1.27E-05 | 5.232 | 4.895255 | up-regulated | 0.646854 | 1.627461 | 5.465313 | 2.80E-07 | 1.43E-06 | 6.197131 | 5.843752 | up-regulated |
| TNFRSF12A | Yes | No | 1.857734 | 5.133022 | 5.86576 | 3.99E-08 | 9.03E-07 | 8.261775 | 6.044417 | up-regulated | 1.574832 | 2.376585 | 8.224436 | 3.76E-13 | 7.23E-12 | 19.42366 | 11.14094 | up-regulated |
| FSCN1 | Yes | No | 1.847559 | 7.129906 | 7.814719 | 2.28E-12 | 2.31E-10 | 17.74872 | 9.635805 | up-regulated | 1.710065 | 3.062945 | 10.35675 | 4.55E-18 | 3.34E-16 | 30.57425 | 15.4764 | up-regulated |
| PLAUR | Yes | No | 1.823269 | 3.59279 | 7.509861 | 1.12E-11 | 8.83E-10 | 16.19321 | 9.053926 | up-regulated | 0.760796 | 1.061792 | 7.150905 | 9.18E-11 | 9.96E-10 | 14.02528 | 9.001848 | up-regulated |
| MMP10 | Yes | Yes | 1.820159 | 4.247587 | 2.600761 | 0.010463 | 0.030561 | -3.50896 | 1.514837 | up-regulated | 1.528853 | 1.632885 | 5.843953 | 5.03E-08 | 3.01E-07 | 7.864195 | 6.520835 | up-regulated |
| VCAN | No | Yes | 1.81551 | 2.780791 | 4.384182 | 2.50E-05 | 0.0002 | 2.086734 | 3.698428 | up-regulated | 0.788675 | 1.470233 | 5.142968 | 1.15E-06 | 5.19E-06 | 4.829211 | 5.284799 | up-regulated |
| AMIGO2 | Yes | No | 1.789853 | 2.61768 | 5.16734 | 9.50E-07 | 1.30E-05 | 5.209104 | 4.886403 | up-regulated | 0.698601 | 1.929181 | 4.431024 | 2.18E-05 | 7.47E-05 | 1.998403 | 4.12694 | up-regulated |
| SLC2A1 | Yes | No | 1.783145 | 7.618244 | 6.977027 | 1.74E-10 | 8.75E-09 | 13.52925 | 8.057983 | up-regulated | 1.489769 | 2.822779 | 9.664691 | 1.85E-16 | 8.12E-15 | 26.92429 | 14.0904 | up-regulated |
| LAMB3 | No | Yes | 1.775367 | 7.273912 | 5.335578 | 4.52E-07 | 6.86E-06 | 5.923185 | 5.1639 | up-regulated | 1.419763 | 2.612905 | 9.477513 | 5.02E-16 | 1.93E-14 | 25.93996 | 13.71456 | up-regulated |
| ZNF185 | Yes | No | -2.17938 | 5.054058 | -7.00371 | 1.52E-10 | 7.82E-09 | 13.66081 | 8.106692 | down-regulated | -0.55957 | 1.78724 | -3.22995 | 0.001622 | 0.003686 | -2.06545 | 2.433388 | down-regulated |
| LCN2 | No | Yes | -2.14402 | 6.696852 | -3.40826 | 0.000889 | 0.004042 | -1.26608 | 2.39344 | down-regulated | -1.58146 | 5.766211 | -6.58338 | 1.51E-09 | 1.26E-08 | 11.28188 | 7.900024 | down-regulated |
| MUC20 | Yes | Yes | -2.08525 | 2.18479 | -5.20091 | 8.20E-07 | 1.14E-05 | 5.350438 | 4.942134 | down-regulated | -1.14941 | 1.797984 | -9.35043 | 9.88E-16 | 3.52E-14 | 25.27276 | 13.45385 | down-regulated |
| PDPN | Yes | No | 1.742798 | 4.575538 | 4.578418 | 1.15E-05 | 0.000104 | 2.828243 | 3.98453 | up-regulated | 0.814389 | 0.980114 | 8.176861 | 4.82E-13 | 9.06E-12 | 19.17992 | 11.04297 | up-regulated |
| C1orf116 | Yes | No | -2.03291 | 4.187662 | -7.00216 | 1.53E-10 | 7.84E-09 | 13.65319 | 8.105547 | down-regulated | -0.91478 | 3.206018 | -4.81979 | 4.52E-06 | 1.80E-05 | 3.509863 | 4.745247 | down-regulated |
| MYO1B | Yes | No | 1.683074 | 4.26287 | 7.434321 | 1.67E-11 | 1.22E-09 | 15.81108 | 8.914034 | up-regulated | 0.771147 | 0.912417 | 10.60015 | 1.24E-18 | 1.12E-16 | 31.85957 | 15.95062 | up-regulated |
| LYZ | No | Yes | -2.01468 | 6.190862 | -3.08177 | 0.002549 | 0.009671 | -2.23508 | 2.014507 | down-regulated | -4.01261 | 7.489199 | -7.68199 | 6.21E-12 | 8.93E-11 | 16.6671 | 10.04919 | down-regulated |
| COL16A1 | Yes | Yes | 1.680385 | 3.297387 | 5.308664 | 5.09E-07 | 7.61E-06 | 5.807997 | 5.118824 | up-regulated | 0.656273 | 1.273117 | 6.040024 | 2.02E-08 | 1.32E-07 | 8.750838 | 6.87965 | up-regulated |
| TGFB1 | No | Yes | 1.67178 | 5.161615 | 9.231648 | 1.10E-15 | 2.86E-13 | 25.19148 | 12.54339 | up-regulated | 0.523153 | 1.179649 | 7.722345 | 5.05E-12 | 7.45E-11 | 16.87031 | 10.12774 | up-regulated |
| LRG1 | No | Yes | -1.99697 | 3.09861 | -4.94272 | 2.51E-06 | 2.89E-05 | 4.278525 | 4.539273 | down-regulated | -1.03376 | 2.370797 | -6.48591 | 2.43E-09 | 1.94E-08 | 10.82043 | 7.712687 | down-regulated |
| NMB | Yes | Yes | 1.654083 | 3.55243 | 5.369901 | 3.88E-07 | 5.99E-06 | 6.070602 | 5.222648 | up-regulated | 0.668884 | 1.073548 | 6.544156 | 1.83E-09 | 1.50E-08 | 11.09582 | 7.824416 | up-regulated |
| C1QTNF6 | No | Yes | 1.619465 | 2.284009 | 6.136485 | 1.11E-08 | 3.15E-07 | 9.503124 | 6.501894 | up-regulated | 0.848511 | 1.067792 | 9.05637 | 4.71E-15 | 1.43E-13 | 23.73317 | 12.84351 | up-regulated |
| CLIC4 | Yes | No | 1.615214 | 5.158407 | 7.558238 | 8.74E-12 | 7.09E-10 | 16.43865 | 9.14936 | up-regulated | 0.531097 | 1.421186 | 7.837683 | 2.79E-12 | 4.39E-11 | 17.4529 | 10.35771 | up-regulated |
| ANKRD35 | Yes | No | -1.94952 | 2.111785 | -6.02705 | 1.86E-08 | 4.80E-07 | 8.99774 | 6.319105 | down-regulated | -0.51353 | 0.982676 | -5.19347 | 9.25E-07 | 4.25E-06 | 5.040223 | 5.371487 | down-regulated |
| CLDN4 | Yes | No | -1.94872 | 5.348523 | -4.55946 | 1.24E-05 | 0.000111 | 2.754866 | 3.954825 | down-regulated | -0.70035 | 4.30694 | -3.49577 | 0.000677 | 0.001674 | -1.25242 | 2.776253 | down-regulated |
| TFRC | No | Yes | 1.61394 | 5.078419 | 4.975076 | 2.19E-06 | 2.57E-05 | 4.410907 | 4.589321 | up-regulated | 1.089378 | 2.219337 | 9.009517 | 6.04E-15 | 1.81E-13 | 23.4885 | 12.74259 | up-regulated |
| PTK6 | Yes | No | -1.92251 | 3.919962 | -6.0312 | 1.83E-08 | 4.73E-07 | 9.016812 | 6.325398 | down-regulated | -0.88495 | 1.873639 | -5.86366 | 4.59E-08 | 2.78E-07 | 7.952606 | 6.555283 | down-regulated |
| PHLDB2 | Yes | No | 1.613527 | 2.882721 | 5.903014 | 3.35E-08 | 7.79E-07 | 8.430808 | 6.108526 | up-regulated | 0.542501 | 0.639302 | 8.814161 | 1.70E-14 | 4.47E-13 | 22.4705 | 12.35 | up-regulated |
| COL5A3 | Yes | Yes | 1.6037 | 3.20276 | 5.031475 | 1.71E-06 | 2.10E-05 | 4.643025 | 4.677085 | up-regulated | 0.549778 | 0.785734 | 6.389333 | 3.86E-09 | 2.93E-08 | 10.36626 | 7.532726 | up-regulated |
| IL33 | No | Yes | -1.87543 | 1.738445 | -5.57489 | 1.53E-07 | 2.78E-06 | 6.962811 | 5.555208 | down-regulated | -0.62648 | 0.7117 | -7.96732 | 1.43E-12 | 2.42E-11 | 18.11067 | 10.61637 | down-regulated |
| ENAH | Yes | No | 1.596136 | 3.506916 | 8.613613 | 3.19E-14 | 5.66E-12 | 21.90989 | 11.24717 | up-regulated | 0.670067 | 0.999963 | 11.69401 | 3.58E-21 | 8.20E-19 | 37.62215 | 18.08637 | up-regulated |
| SLPI | No | Yes | -1.82054 | 8.843747 | -2.98131 | 0.003472 | 0.012388 | -2.5166 | 1.907004 | down-regulated | -2.38289 | 7.91577 | -6.8186 | 4.79E-10 | 4.42E-09 | 12.40775 | 8.354282 | down-regulated |
| GPRIN1 | Yes | No | 1.582259 | 2.166328 | 8.715791 | 1.83E-14 | 3.58E-12 | 22.44931 | 11.44633 | up-regulated | 0.612572 | 0.469321 | 10.85523 | 3.16E-19 | 3.40E-17 | 33.20616 | 16.46799 | up-regulated |
| TNNI2 | Yes | No | -1.73021 | 2.298497 | -2.90629 | 0.004352 | 0.014926 | -2.72159 | 1.826042 | down-regulated | -0.90048 | 1.801458 | -3.7312 | 0.0003 | 0.000798 | -0.48945 | 3.097894 | down-regulated |
| AGRN | Yes | Yes | 1.571667 | 5.223684 | 6.05798 | 1.61E-08 | 4.24E-07 | 9.140099 | 6.372131 | up-regulated | 0.784888 | 2.393932 | 6.849513 | 4.11E-10 | 3.85E-09 | 12.55691 | 8.414006 | up-regulated |
| CSPG4 | Yes | No | 1.559067 | 3.078006 | 3.456445 | 0.000756 | 0.003523 | -1.11622 | 2.453045 | up-regulated | 0.661213 | 1.007451 | 5.700135 | 9.72E-08 | 5.48E-07 | 7.223745 | 6.261491 | up-regulated |
| LY6K | Yes | Yes | 1.549468 | 2.591256 | 4.971114 | 2.22E-06 | 2.61E-05 | 4.394663 | 4.58299 | up-regulated | 1.17917 | 1.315913 | 8.194372 | 4.40E-13 | 8.36E-12 | 19.2696 | 11.07776 | up-regulated |
| MSN | Yes | No | 1.547413 | 6.649603 | 6.593394 | 1.19E-09 | 4.55E-08 | 11.66191 | 7.342076 | up-regulated | 0.698148 | 1.579788 | 9.073742 | 4.30E-15 | 1.33E-13 | 23.82394 | 12.87644 | up-regulated |
| GALNT12 | Yes | No | -1.69759 | 2.234429 | -6.7081 | 6.73E-10 | 2.78E-08 | 12.21533 | 7.555638 | down-regulated | -0.51093 | 0.55768 | -13.4982 | 2.70E-25 | 2.81E-22 | 46.97773 | 21.55095 | down-regulated |
| UBE2S | Yes | No | 1.536528 | 3.868935 | 8.857391 | 8.50E-15 | 1.86E-12 | 23.19906 | 11.73002 | up-regulated | 1.059328 | 2.455735 | 9.134942 | 3.11E-15 | 9.83E-14 | 24.1439 | 13.0074 | up-regulated |
| MCAM | Yes | No | 1.526247 | 3.646587 | 6.684845 | 7.56E-10 | 3.07E-08 | 12.1028 | 7.512507 | up-regulated | 0.627195 | 2.804933 | 5.198645 | 9.04E-07 | 4.17E-06 | 5.0619 | 5.380114 | up-regulated |
| SLC7A5 | Yes | No | 1.512835 | 6.646481 | 4.432103 | 2.07E-05 | 0.000171 | 2.267547 | 3.767695 | up-regulated | 1.305135 | 2.170029 | 8.322539 | 2.25E-13 | 4.58E-12 | 19.92735 | 11.33958 | up-regulated |
| CLU | No | Yes | -1.69691 | 4.932435 | -3.58858 | 0.000481 | 0.002423 | -0.69652 | 2.615603 | down-regulated | -2.23719 | 3.63979 | -10.8611 | 3.06E-19 | 3.33E-17 | 33.23714 | 16.47794 | down-regulated |
| ICAM1 | Yes | No | 1.49879 | 4.101871 | 4.223442 | 4.69E-05 | 0.000341 | 1.4907 | 3.466638 | up-regulated | 0.554327 | 2.167217 | 3.279177 | 0.001385 | 0.003196 | -1.91887 | 2.495448 | up-regulated |
| NUCB2 | No | Yes | -1.68285 | 2.714046 | -9.54033 | 2.03E-16 | 6.89E-14 | 26.84445 | 13.16159 | down-regulated | -0.82056 | 0.925751 | -11.579 | 6.61E-21 | 1.33E-18 | 37.01821 | 17.87501 | down-regulated |
| CLEC11A | No | Yes | 1.496837 | 2.672379 | 5.761503 | 6.49E-08 | 1.36E-06 | 7.79189 | 5.867273 | up-regulated | 0.614021 | 1.344204 | 6.148165 | 1.22E-08 | 8.29E-08 | 9.246207 | 7.081312 | up-regulated |
| UBE2C | Yes | No | 1.49617 | 5.108413 | 7.06496 | 1.11E-10 | 6.05E-09 | 13.96358 | 8.21823 | up-regulated | 1.537524 | 2.074172 | 11.55387 | 7.55E-21 | 1.49E-18 | 36.88626 | 17.82613 | up-regulated |
| FLNA | Yes | No | 1.476187 | 6.904996 | 6.271116 | 5.78E-09 | 1.80E-07 | 10.13126 | 6.744313 | up-regulated | 0.898875 | 5.064062 | 8.238523 | 3.49E-13 | 6.80E-12 | 19.49591 | 11.16747 | up-regulated |
| TFF3 | No | Yes | -1.67794 | 2.422089 | -2.66508 | 0.008749 | 0.026364 | -3.34962 | 1.578995 | down-regulated | -2.38511 | 2.140942 | -7.97885 | 1.35E-12 | 2.30E-11 | 18.16934 | 10.63836 | down-regulated |
| SPHK1 | Yes | No | 1.4365 | 3.836962 | 6.388836 | 3.26E-09 | 1.08E-07 | 10.68607 | 6.96611 | up-regulated | 0.711811 | 1.48195 | 7.799847 | 3.39E-12 | 5.23E-11 | 17.26151 | 10.28138 | up-regulated |
| LOX | No | Yes | 1.432136 | 2.773907 | 4.401155 | 2.34E-05 | 0.00019 | 2.150614 | 3.721972 | up-regulated | 0.836038 | 1.625042 | 6.776362 | 5.89E-10 | 5.32E-09 | 12.20434 | 8.273739 | up-regulated |
| SLC16A1 | Yes | No | 1.425445 | 4.59808 | 5.968998 | 2.45E-08 | 6.02E-07 | 8.731617 | 6.22058 | up-regulated | 1.134551 | 1.205881 | 10.59425 | 1.28E-18 | 1.14E-16 | 31.82842 | 15.94281 | up-regulated |
| CEP55 | Yes | No | 1.422874 | 3.85122 | 6.362134 | 3.71E-09 | 1.21E-07 | 10.55978 | 6.916756 | up-regulated | 0.812697 | 0.810569 | 11.03032 | 1.24E-19 | 1.50E-17 | 34.12979 | 16.8246 | up-regulated |
| CDH3 | Yes | No | 1.398245 | 5.858338 | 4.396896 | 2.38E-05 | 0.000192 | 2.134568 | 3.715708 | up-regulated | 1.11065 | 1.387633 | 9.165187 | 2.65E-15 | 8.62E-14 | 24.30214 | 13.06453 | up-regulated |
| HLA-A | Yes | No | 1.381103 | 8.704172 | 5.117463 | 1.18E-06 | 1.54E-05 | 5.000173 | 4.812701 | up-regulated | 0.568272 | 6.304238 | 3.527215 | 0.000608 | 0.00152 | -1.1528 | 2.818159 | up-regulated |
| ATP1B3 | Yes | No | 1.362692 | 7.4438 | 6.855368 | 3.22E-10 | 1.49E-08 | 12.9321 | 7.828164 | up-regulated | 1.045696 | 2.285114 | 9.461002 | 5.48E-16 | 2.08E-14 | 25.85323 | 13.68164 | up-regulated |
| SORT1 | Yes | No | -1.58349 | 3.169442 | -7.74386 | 3.31E-12 | 3.19E-10 | 17.38536 | 9.49562 | down-regulated | -0.63858 | 1.334352 | -8.50347 | 8.73E-14 | 1.95E-12 | 20.85981 | 11.70984 | down-regulated |
| CHI3L1 | No | Yes | 1.352203 | 3.237652 | 2.92523 | 0.004112 | 0.014237 | -2.67025 | 1.846576 | up-regulated | 0.955644 | 1.479919 | 5.21013 | 8.60E-07 | 3.98E-06 | 5.110091 | 5.399922 | up-regulated |
| EPHA2 | Yes | No | -1.47613 | 5.277404 | -5.54525 | 1.76E-07 | 3.13E-06 | 6.832552 | 5.504023 | down-regulated | -0.55701 | 2.362953 | -3.81496 | 0.000223 | 0.000606 | -0.20866 | 3.217399 | down-regulated |
| SLC3A2 | Yes | No | 1.35069 | 6.100288 | 6.891529 | 2.68E-10 | 1.26E-08 | 13.10913 | 7.90014 | up-regulated | 0.838911 | 1.897354 | 9.989372 | 3.26E-17 | 1.82E-15 | 28.6352 | 14.74021 | up-regulated |
| CGN | Yes | No | -1.46477 | 2.11908 | -4.99046 | 2.05E-06 | 2.44E-05 | 4.474072 | 4.612482 | down-regulated | -0.64463 | 1.236703 | -8.12262 | 6.39E-13 | 1.17E-11 | 18.90245 | 10.93242 | down-regulated |
| CTSV | Yes | No | 1.310247 | 3.60307 | 3.669009 | 0.000364 | 0.001927 | -0.43487 | 2.715068 | up-regulated | 1.542285 | 2.013557 | 8.949105 | 8.32E-15 | 2.40E-13 | 23.17331 | 12.62056 | up-regulated |
| EFNB1 | Yes | No | 1.304144 | 5.622662 | 5.997938 | 2.14E-08 | 5.37E-07 | 8.864114 | 6.270275 | up-regulated | 1.012484 | 2.398676 | 8.962 | 7.77E-15 | 2.26E-13 | 23.24056 | 12.64553 | up-regulated |
| APOD | Yes | Yes | -1.45776 | 2.866123 | -3.48119 | 0.000695 | 0.003292 | -1.0386 | 2.482559 | down-regulated | -2.14076 | 2.092579 | -11.882 | 1.32E-21 | 3.52E-19 | 38.60795 | 18.45299 | down-regulated |
| UCN2 | No | Yes | 1.292483 | 1.806692 | 4.993444 | 2.02E-06 | 2.42E-05 | 4.486313 | 4.61698 | up-regulated | 1.289717 | 1.353897 | 10.26249 | 7.55E-18 | 5.17E-16 | 30.07659 | 15.28665 | up-regulated |
| CXCL5 | No | Yes | 1.291734 | 1.310987 | 3.669306 | 0.000363 | 0.001926 | -0.43389 | 2.715404 | up-regulated | 1.741583 | 1.377305 | 7.356056 | 3.27E-11 | 4.00E-10 | 15.03813 | 9.398243 | up-regulated |
| IGFBP3 | No | Yes | 1.287875 | 5.858853 | 3.188409 | 0.001822 | 0.007336 | -1.9276 | 2.134526 | up-regulated | 1.343195 | 3.777408 | 6.90354 | 3.15E-10 | 3.03E-09 | 12.8183 | 8.518752 | up-regulated |
| PPP1R18 | Yes | No | 1.281963 | 4.452781 | 6.68059 | 7.72E-10 | 3.13E-08 | 12.08223 | 7.505087 | up-regulated | 0.559541 | 2.793149 | 6.078039 | 1.69E-08 | 1.12E-07 | 8.924473 | 6.949587 | up-regulated |
| ITGB4 | Yes | No | 1.276867 | 6.796262 | 5.450226 | 2.70E-07 | 4.43E-06 | 6.417826 | 5.353513 | up-regulated | 0.713483 | 3.34735 | 6.607523 | 1.35E-09 | 1.13E-08 | 11.39667 | 7.946177 | up-regulated |
| ITGA3 | Yes | No | 1.268541 | 4.974578 | 3.937787 | 0.000138 | 0.000855 | 0.472621 | 3.067835 | up-regulated | 1.063416 | 2.135028 | 7.613853 | 8.81E-12 | 1.22E-10 | 16.32467 | 9.913722 | up-regulated |
| MIF | No | Yes | 1.256543 | 5.606863 | 5.45098 | 2.69E-07 | 4.43E-06 | 6.421102 | 5.353906 | up-regulated | 0.723575 | 4.161247 | 5.267575 | 6.70E-07 | 3.17E-06 | 5.352092 | 5.498679 | up-regulated |
| CAST | Yes | No | -1.36273 | 4.614378 | -8.14542 | 3.94E-13 | 5.08E-11 | 19.45782 | 10.29441 | down-regulated | -0.51 | 1.425905 | -6.48271 | 2.46E-09 | 1.96E-08 | 10.80532 | 7.707079 | down-regulated |
| CSF2 | No | Yes | 1.185158 | 1.217913 | 3.018914 | 0.003095 | 0.011277 | -2.41217 | 1.947797 | up-regulated | 0.601766 | 0.36301 | 5.540121 | 2.00E-07 | 1.06E-06 | 6.521533 | 5.976514 | up-regulated |
| CLDN23 | Yes | No | -1.34897 | 1.318435 | -6.67919 | 7.78E-10 | 3.14E-08 | 12.07544 | 7.502941 | down-regulated | -0.97519 | 1.716047 | -8.52099 | 7.96E-14 | 1.79E-12 | 20.95036 | 11.74669 | down-regulated |
| TACSTD2 | Yes | No | -1.33077 | 8.186782 | -4.84037 | 3.87E-06 | 4.21E-05 | 3.863476 | 4.375715 | down-regulated | -0.56574 | 7.727338 | -3.10233 | 0.002427 | 0.005311 | -2.43687 | 2.274831 | down-regulated |
| ACTN1 | Yes | No | 1.156836 | 5.054933 | 4.750692 | 5.64E-06 | 5.73E-05 | 3.504652 | 4.242121 | up-regulated | 0.634239 | 1.432766 | 6.741961 | 6.98E-10 | 6.22E-09 | 12.03908 | 8.206198 | up-regulated |
| ERVMER34-1 | No | Yes | 1.128316 | 1.813481 | 4.434069 | 2.05E-05 | 0.00017 | 2.274995 | 3.770736 | up-regulated | 0.728124 | 0.717155 | 8.179888 | 4.74E-13 | 8.93E-12 | 19.19542 | 11.04894 | up-regulated |
| FCGR2A | Yes | No | 1.120772 | 2.344094 | 4.390359 | 2.44E-05 | 0.000196 | 2.10996 | 3.707465 | up-regulated | 0.872773 | 2.116499 | 6.953261 | 2.46E-10 | 2.43E-09 | 13.05958 | 8.614483 | up-regulated |
| SLC39A6 | Yes | No | 1.111572 | 4.893201 | 5.294164 | 5.43E-07 | 8.03E-06 | 5.74609 | 5.095105 | up-regulated | 0.818517 | 2.41237 | 9.648436 | 2.02E-16 | 8.69E-15 | 26.83874 | 14.06076 | up-regulated |
| STEAP4 | Yes | No | -1.25406 | 1.758353 | -3.66778 | 0.000365 | 0.001934 | -0.43891 | 2.713652 | down-regulated | -0.87537 | 1.739868 | -7.06269 | 1.43E-10 | 1.48E-09 | 13.59297 | 8.829172 | down-regulated |
| SLC38A5 | Yes | No | 1.072934 | 3.250714 | 2.802225 | 0.005913 | 0.019172 | -2.99838 | 1.717322 | up-regulated | 0.648258 | 1.046265 | 6.301137 | 5.89E-09 | 4.29E-08 | 9.954269 | 7.368011 | up-regulated |
| FZD6 | Yes | No | 1.060752 | 4.797649 | 4.648909 | 8.59E-06 | 8.12E-05 | 3.102931 | 4.09019 | up-regulated | 0.828709 | 2.004682 | 9.829791 | 7.65E-17 | 3.80E-15 | 27.79382 | 14.42064 | up-regulated |
| ERBB3 | Yes | Yes | -1.23002 | 3.507456 | -5.74476 | 7.02E-08 | 1.45E-06 | 7.716885 | 5.839009 | down-regulated | -0.59622 | 2.681947 | -6.34156 | 4.86E-09 | 3.60E-08 | 10.14274 | 7.44337 | down-regulated |
| ALOXE3 | Yes | No | 1.047029 | 1.582275 | 3.270285 | 0.0014 | 0.005912 | -1.68553 | 2.228265 | up-regulated | 0.512799 | 0.322943 | 7.158329 | 8.84E-11 | 9.64E-10 | 14.06175 | 9.015995 | up-regulated |
| FUT2 | Yes | No | -1.21822 | 3.195997 | -4.04098 | 9.40E-05 | 0.000617 | 0.834192 | 3.20949 | down-regulated | -1.03843 | 2.458527 | -9.22881 | 1.89E-15 | 6.39E-14 | 24.63523 | 13.19449 | down-regulated |
| KNSTRN | Yes | No | 1.037384 | 2.801733 | 6.843102 | 3.42E-10 | 1.57E-08 | 12.87214 | 7.804392 | up-regulated | 0.743166 | 1.175319 | 10.80089 | 4.22E-19 | 4.44E-17 | 32.91934 | 16.35215 | up-regulated |
| ATP13A3 | Yes | No | 1.023313 | 3.61067 | 4.532928 | 1.38E-05 | 0.000121 | 2.652541 | 3.918665 | up-regulated | 0.714872 | 1.645116 | 10.5998 | 1.24E-18 | 1.12E-16 | 31.85773 | 15.95062 | up-regulated |
| CDCA4 | Yes | No | 1.021836 | 4.271099 | 6.001862 | 2.10E-08 | 5.28E-07 | 8.882107 | 6.277252 | up-regulated | 0.827899 | 1.403629 | 11.31321 | 2.73E-20 | 4.37E-18 | 35.62045 | 17.35903 | up-regulated |
| AP2M1 | Yes | No | 1.014541 | 6.82746 | 7.239245 | 4.56E-11 | 2.81E-09 | 14.83084 | 8.551525 | up-regulated | 0.782205 | 4.295906 | 9.214758 | 2.03E-15 | 6.86E-14 | 24.56163 | 13.1636 | up-regulated |
| JAG1 | Yes | No | 1.001214 | 5.276548 | 4.259584 | 4.08E-05 | 0.000304 | 1.623296 | 3.516717 | up-regulated | 0.81171 | 3.150722 | 6.633401 | 1.19E-09 | 1.01E-08 | 11.51989 | 7.996411 | up-regulated |
| ULBP2 | Yes | Yes | 0.997226 | 3.220805 | 3.134014 | 0.002165 | 0.008445 | -2.08556 | 2.073397 | up-regulated | 0.803023 | 0.834383 | 8.806026 | 1.77E-14 | 4.64E-13 | 22.4282 | 12.33316 | up-regulated |
| FHL1 | Yes | No | -1.21501 | 2.526025 | -2.7147 | 0.007605 | 0.023551 | -3.22435 | 1.627997 | down-regulated | -0.55047 | 0.791646 | -5.28446 | 6.22E-07 | 2.96E-06 | 5.423517 | 5.528117 | down-regulated |
| GPC1 | Yes | No | 0.982356 | 6.04268 | 3.825503 | 0.000208 | 0.001218 | 0.087411 | 2.91446 | up-regulated | 0.510728 | 2.309956 | 4.911616 | 3.08E-06 | 1.27E-05 | 3.879164 | 4.89684 | up-regulated |
| SFRP1 | No | Yes | -1.21267 | 1.979208 | -2.99278 | 0.003353 | 0.012039 | -2.48486 | 1.919405 | down-regulated | -0.65444 | 0.819689 | -7.84824 | 2.64E-12 | 4.19E-11 | 17.50635 | 10.37768 | down-regulated |
| ABCC5 | Yes | No | 0.970207 | 3.691648 | 2.6565 | 0.008962 | 0.026878 | -3.37108 | 1.570606 | up-regulated | 0.518493 | 1.475967 | 4.357127 | 2.92E-05 | 9.71E-05 | 1.721065 | 4.012994 | up-regulated |
| PRC1 | Yes | No | 0.959662 | 3.683612 | 4.88643 | 3.19E-06 | 3.54E-05 | 4.049546 | 4.451276 | up-regulated | 0.910853 | 1.287984 | 10.72678 | 6.28E-19 | 6.14E-17 | 32.52816 | 16.21211 | up-regulated |
| STARD4 | Yes | No | 0.957258 | 2.06859 | 5.107481 | 1.23E-06 | 1.59E-05 | 4.958512 | 4.798121 | up-regulated | 0.540112 | 1.26152 | 7.97625 | 1.36E-12 | 2.32E-11 | 18.15608 | 10.63408 | up-regulated |
| PLIN4 | Yes | No | -1.18832 | 0.743706 | -5.4376 | 2.86E-07 | 4.64E-06 | 6.363039 | 5.333258 | down-regulated | -0.65721 | 0.775326 | -5.52669 | 2.13E-07 | 1.12E-06 | 6.463108 | 5.952428 | down-regulated |
| AHNAK | Yes | No | -1.17514 | 6.405671 | -4.70106 | 6.93E-06 | 6.81E-05 | 3.308008 | 4.167148 | down-regulated | -0.58819 | 5.46782 | -5.14879 | 1.12E-06 | 5.07E-06 | 4.85348 | 5.294751 | down-regulated |
| OGN | No | Yes | -1.14363 | 0.783829 | -4.2661 | 3.97E-05 | 0.000298 | 1.647301 | 3.526289 | down-regulated | -0.56763 | 1.188673 | -4.54523 | 1.39E-05 | 4.95E-05 | 2.4334 | 4.305464 | down-regulated |
| OLFML2A | No | Yes | 0.956583 | 3.427125 | 3.240489 | 0.001542 | 0.006404 | -1.77422 | 2.193516 | up-regulated | 0.522952 | 1.33565 | 6.057032 | 1.87E-08 | 1.23E-07 | 8.828454 | 6.910916 | up-regulated |
| TMC5 | Yes | No | -1.13465 | 1.178786 | -3.37771 | 0.000984 | 0.004403 | -1.36018 | 2.356229 | down-regulated | -0.65289 | 0.614148 | -6.4739 | 2.57E-09 | 2.04E-08 | 10.7638 | 7.689907 | down-regulated |
| GPRC5A | Yes | No | -1.07095 | 3.525735 | -2.60558 | 0.010325 | 0.030233 | -3.49714 | 1.519517 | down-regulated | -0.51384 | 2.062557 | -3.39365 | 0.000952 | 0.002273 | -1.57095 | 2.643355 | down-regulated |
| FOLR1 | No | Yes | -1.06728 | 1.213469 | -2.63213 | 0.009592 | 0.028418 | -3.43168 | 1.5464 | down-regulated | -1.58482 | 1.223953 | -7.30312 | 4.27E-11 | 5.03E-10 | 14.77579 | 9.298197 | down-regulated |
| NME1 | Yes | No | 0.942488 | 4.107644 | 5.464651 | 2.53E-07 | 4.23E-06 | 6.480508 | 5.37414 | up-regulated | 0.669202 | 1.698411 | 8.534749 | 7.41E-14 | 1.68E-12 | 21.02146 | 11.77547 | up-regulated |
| MMRN1 | No | Yes | -1.05553 | 0.709674 | -5.54254 | 1.78E-07 | 3.16E-06 | 6.820687 | 5.499851 | down-regulated | -0.69309 | 0.512027 | -10.1043 | 1.76E-17 | 1.07E-15 | 29.24159 | 14.9724 | down-regulated |
| CD109 | Yes | No | 0.924751 | 3.939852 | 3.463809 | 0.000738 | 0.003458 | -1.09317 | 2.461165 | up-regulated | 0.625757 | 0.998427 | 8.23814 | 3.50E-13 | 6.81E-12 | 19.49394 | 11.16705 | up-regulated |
| ZNF598 | Yes | No | 0.91915 | 4.094769 | 6.734517 | 5.90E-10 | 2.50E-08 | 12.34341 | 7.602813 | up-regulated | 0.558396 | 2.112357 | 7.905839 | 1.96E-12 | 3.22E-11 | 17.79834 | 10.49221 | up-regulated |
| OAS3 | Yes | No | 0.897993 | 4.207185 | 2.940023 | 0.003934 | 0.01372 | -2.62997 | 1.862655 | up-regulated | 1.024901 | 2.315844 | 6.562439 | 1.68E-09 | 1.38E-08 | 11.18249 | 7.86033 | up-regulated |
| PKP1 | Yes | No | 0.847462 | 8.035047 | 3.66682 | 0.000366 | 0.001939 | -0.44205 | 2.712425 | up-regulated | 1.36809 | 3.644202 | 5.852588 | 4.84E-08 | 2.91E-07 | 7.902921 | 6.535482 | up-regulated |
| FLAD1 | Yes | No | 0.826295 | 3.702617 | 6.437511 | 2.57E-09 | 8.83E-08 | 10.91694 | 7.054279 | up-regulated | 0.524571 | 1.77892 | 10.33938 | 5.00E-18 | 3.64E-16 | 30.48257 | 15.43936 | up-regulated |
| STAT2 | Yes | No | 0.809404 | 4.231404 | 4.084306 | 7.98E-05 | 0.000538 | 0.988104 | 3.269521 | up-regulated | 0.515388 | 3.175111 | 5.394482 | 3.83E-07 | 1.91E-06 | 5.89233 | 5.720011 | up-regulated |
| MYH9 | Yes | No | 0.808335 | 7.678527 | 4.08922 | 7.84E-05 | 0.00053 | 1.00564 | 3.276124 | up-regulated | 0.588207 | 3.728813 | 8.481022 | 9.82E-14 | 2.16E-12 | 20.74389 | 11.66586 | up-regulated |
| ADH1B | Yes | No | -1.0045 | 0.231017 | -8.30461 | 1.68E-13 | 2.46E-11 | 20.28764 | 10.60973 | down-regulated | -1.3739 | 0.809513 | -11.3405 | 2.36E-20 | 3.86E-18 | 35.76409 | 17.41351 | down-regulated |
| ITM2A | Yes | No | -0.9886 | 2.102555 | -3.88371 | 0.000168 | 0.001016 | 0.286036 | 2.993176 | down-regulated | -0.91378 | 1.359505 | -10.0772 | 2.03E-17 | 1.23E-15 | 29.09871 | 14.91085 | down-regulated |
| IFITM1 | Yes | No | 0.807302 | 6.49857 | 2.461479 | 0.015248 | 0.041415 | -3.84205 | 1.382841 | up-regulated | 0.759299 | 4.842972 | 4.50908 | 1.60E-05 | 5.64E-05 | 2.294891 | 4.248491 | up-regulated |
| STIP1 | Yes | No | 0.804559 | 5.583763 | 5.542558 | 1.78E-07 | 3.16E-06 | 6.820755 | 5.499851 | up-regulated | 0.791647 | 2.664289 | 8.558662 | 6.53E-14 | 1.50E-12 | 21.14512 | 11.82371 | up-regulated |
| LGALS3 | Yes | Yes | -0.97903 | 6.422683 | -4.13551 | 6.57E-05 | 0.000455 | 1.171612 | 3.341799 | down-regulated | -0.69257 | 2.993721 | -6.12133 | 1.38E-08 | 9.31E-08 | 9.122862 | 7.031026 | down-regulated |
| BMP2 | No | Yes | 0.798347 | 2.468858 | 2.487288 | 0.014236 | 0.039278 | -3.78157 | 1.405854 | up-regulated | 0.680715 | 1.093755 | 6.10939 | 1.46E-08 | 9.81E-08 | 9.068079 | 7.008174 | up-regulated |
| PI15 | No | Yes | 0.771344 | 1.080739 | 3.43555 | 0.000811 | 0.003734 | -1.18142 | 2.427802 | up-regulated | 0.706518 | 1.042895 | 8.756983 | 2.30E-14 | 5.82E-13 | 22.17327 | 12.23499 | up-regulated |
| CCT3 | Yes | No | 0.764691 | 6.353207 | 7.479006 | 1.32E-11 | 1.01E-09 | 16.03696 | 8.994467 | up-regulated | 0.567929 | 1.720087 | 8.415338 | 1.39E-13 | 2.94E-12 | 20.40506 | 11.53226 | up-regulated |
| HDGF | No | Yes | 0.729044 | 6.871223 | 5.697776 | 8.73E-08 | 1.73E-06 | 7.507006 | 5.762072 | up-regulated | 0.776373 | 4.381266 | 9.446367 | 5.92E-16 | 2.22E-14 | 25.77636 | 13.653 | up-regulated |
| CFH | No | Yes | -0.88107 | 2.927738 | -3.09829 | 0.002421 | 0.009263 | -2.18804 | 2.033245 | down-regulated | -0.86408 | 1.711407 | -8.5755 | 5.98E-14 | 1.38E-12 | 21.23224 | 11.85896 | down-regulated |
| ITGB1 | Yes | No | 0.725702 | 5.451965 | 2.433433 | 0.016421 | 0.043891 | -3.90712 | 1.357628 | up-regulated | 0.688973 | 2.483764 | 7.323077 | 3.86E-11 | 4.60E-10 | 14.87463 | 9.336914 | up-regulated |
| ERBB2 | Yes | No | -0.88017 | 4.219898 | -3.39356 | 0.000934 | 0.004205 | -1.31145 | 2.376233 | down-regulated | -0.59801 | 2.140375 | -5.12791 | 1.23E-06 | 5.50E-06 | 4.766533 | 5.259626 | down-regulated |
| CRIP2 | Yes | No | -0.81994 | 4.029737 | -3.05112 | 0.002803 | 0.010427 | -2.32183 | 1.981841 | down-regulated | -0.69381 | 2.610269 | -4.95686 | 2.55E-06 | 1.07E-05 | 4.062789 | 4.971705 | down-regulated |
| ASPM | Yes | No | 0.715897 | 2.195222 | 3.620318 | 0.000431 | 0.002212 | -0.59383 | 2.655188 | up-regulated | 0.900783 | 1.204739 | 9.634196 | 2.17E-16 | 9.25E-15 | 26.7638 | 14.03368 | up-regulated |
| ADRM1 | Yes | No | 0.63717 | 6.110856 | 5.919848 | 3.10E-08 | 7.24E-07 | 8.507376 | 6.140376 | up-regulated | 0.567593 | 3.325049 | 8.275089 | 2.89E-13 | 5.74E-12 | 19.68356 | 11.24123 | up-regulated |
| GLO1 | Yes | No | 0.633115 | 5.798695 | 4.522992 | 1.44E-05 | 0.000125 | 2.61433 | 3.903145 | up-regulated | 0.529784 | 1.954355 | 7.486462 | 1.69E-11 | 2.19E-10 | 15.6871 | 9.660452 | up-regulated |
| C1orf68 | Yes | No | 0.615189 | 0.586361 | 2.45551 | 0.015492 | 0.041954 | -3.85596 | 1.377232 | up-regulated | 0.800909 | 0.511053 | 5.726373 | 8.63E-08 | 4.93E-07 | 7.339942 | 6.307472 | up-regulated |
| OXGR1 | Yes | No | -0.77997 | 0.682662 | -3.98947 | 0.000114 | 0.000728 | 0.6528 | 3.137701 | down-regulated | -1.02403 | 0.828329 | -6.87441 | 3.63E-10 | 3.45E-09 | 12.67727 | 8.46238 | down-regulated |
| GLUL | Yes | No | -0.7744 | 5.896186 | -2.37079 | 0.019332 | 0.049978 | -4.05003 | 1.301222 | down-regulated | -0.71571 | 5.997111 | -5.14519 | 1.14E-06 | 5.14E-06 | 4.838473 | 5.288633 | down-regulated |
| PRRT3 | Yes | No | -0.63654 | 0.787289 | -5.50895 | 2.07E-07 | 3.58E-06 | 6.673626 | 5.445524 | down-regulated | -0.57809 | 1.006421 | -10.8458 | 3.32E-19 | 3.56E-17 | 33.15624 | 16.44849 | down-regulated |
| C1orf74 | Yes | No | 0.598263 | 2.202941 | 2.407428 | 0.017578 | 0.046313 | -3.96685 | 1.334297 | up-regulated | 0.58706 | 2.450889 | 4.077161 | 8.52E-05 | 0.000255 | 0.700898 | 3.594207 | up-regulated |
| GSN | No | Yes | -0.61529 | 5.77554 | -2.73401 | 0.007198 | 0.02252 | -3.17504 | 1.647439 | down-regulated | -0.81066 | 2.136106 | -8.47587 | 1.01E-13 | 2.21E-12 | 20.71732 | 11.65616 | down-regulated |
| SLC38A2 | Yes | No | 0.592992 | 6.025064 | 2.918939 | 0.00419 | 0.014467 | -2.68733 | 1.839616 | up-regulated | 0.644648 | 5.107933 | 5.223203 | 8.13E-07 | 3.78E-06 | 5.165024 | 5.422404 | up-regulated |
| ENO1 | Yes | No | 0.573402 | 9.067761 | 3.517042 | 0.000616 | 0.002986 | -0.92534 | 2.524983 | up-regulated | 0.887027 | 4.834398 | 6.66794 | 1.00E-09 | 8.62E-09 | 11.68468 | 8.064254 | up-regulated |
| YBX1 | Yes | No | 0.565767 | 7.939612 | 4.856249 | 3.62E-06 | 3.97E-05 | 3.927501 | 4.40071 | up-regulated | 0.550417 | 4.170031 | 6.851601 | 4.07E-10 | 3.82E-09 | 12.567 | 8.417831 | up-regulated |
| HMGB2 | No | Yes | 0.56352 | 5.05243 | 2.605731 | 0.01032 | 0.030225 | -3.49677 | 1.519632 | up-regulated | 0.575755 | 4.22006 | 4.607599 | 1.08E-05 | 3.94E-05 | 2.674201 | 4.404439 | up-regulated |
| GAS2L1 | Yes | No | 0.562865 | 3.777593 | 3.638714 | 0.000404 | 0.002099 | -0.53397 | 2.677918 | up-regulated | 0.503398 | 2.126631 | 6.010884 | 2.32E-08 | 1.49E-07 | 8.618112 | 6.82632 | up-regulated |
| BICD2 | Yes | No | 0.559991 | 5.000761 | 2.838104 | 0.005325 | 0.017574 | -2.90394 | 1.755122 | up-regulated | 0.753582 | 2.05226 | 7.728424 | 4.90E-12 | 7.25E-11 | 16.90095 | 10.13944 | up-regulated |
| HP | No | Yes | -0.58736 | 0.341145 | -3.25965 | 0.001449 | 0.006089 | -1.71728 | 2.215424 | down-regulated | -0.61501 | 0.740406 | -3.97304 | 0.000125 | 0.000361 | 0.33425 | 3.442658 | down-regulated |
| ZMYND10 | Yes | No | -0.57051 | 0.343737 | -2.77711 | 0.00636 | 0.020369 | -3.06387 | 1.691034 | down-regulated | -0.94257 | 0.864614 | -5.79091 | 6.42E-08 | 3.77E-07 | 7.626969 | 6.424096 | down-regulated |
| IL6ST | Yes | Yes | -0.52023 | 3.382509 | -2.43086 | 0.016532 | 0.044093 | -3.91306 | 1.35563 | down-regulated | -0.56063 | 2.503739 | -6.82698 | 4.59E-10 | 4.25E-09 | 12.44816 | 8.371357 | down-regulated |
| DNAJC9 | Yes | No | 0.510288 | 2.712395 | 3.733258 | 0.00029 | 0.001597 | -0.22253 | 2.796615 | up-regulated | 0.540662 | 1.994264 | 9.008357 | 6.08E-15 | 1.82E-13 | 23.48244 | 12.74061 | up-regulated |
