# Supplementary material for: Identification of Significant Secreted or Membrane-Located Proteins in Laryngeal Squamous Cell Carcinoma
Source: J Immunol Res. 2022 May 23;2022:9089397. doi: 10.1155/2022/9089397 (PMC9153386; doi:10.1155/2022/9089397)
Supplement: Supplementary 2 — Table S2: survival analyses of differentially expressed SMPs. [file 9089397.f2.docx]

Table S2. Survival analyses of differentially expressed SMPs

| gene | cut.point | p.value | HR | low95 | up95 | group |
| --- | --- | --- | --- | --- | --- | --- |
| MMP1 | 8.606868 | 7.13E-05 | 5.290665 | 2.325333 | 12.03747 | worse |
| KLHDC7B | 1.720753 | 0.000297 | 0.321036 | 0.173475 | 0.594116 | better |
| LY6K | 1.714676 | 0.001615 | 0.350349 | 0.182546 | 0.672404 | better |
| ZNF598 | 4.331473 | 0.002074 | 0.302974 | 0.141687 | 0.64786 | better |
| MMP3 | 6.377719 | 0.002329 | 3.137439 | 1.502875 | 6.549797 | worse |
| LAMA3 | 5.871087 | 0.002334 | 3.123745 | 1.500272 | 6.50401 | worse |
| STC2 | 1.98623 | 0.004224 | 2.480816 | 1.331277 | 4.622965 | worse |
| SLC38A5 | 3.044764 | 0.004345 | 0.42613 | 0.23711 | 0.765832 | better |
| ATP1B3 | 8.325671 | 0.005659 | 2.815482 | 1.352419 | 5.861304 | worse |
| LAMC2 | 8.370351 | 0.006147 | 2.464747 | 1.292757 | 4.699241 | worse |
| FSCN1 | 7.752151 | 0.006495 | 2.277364 | 1.259025 | 4.119368 | worse |
| CDH3 | 6.51702 | 0.006565 | 2.260346 | 1.255424 | 4.069672 | worse |
| LOX | 2.272538 | 0.006571 | 3.051353 | 1.364893 | 6.8216 | worse |
| FST | 4.091488 | 0.006781 | 2.734781 | 1.320146 | 5.665301 | worse |
| ITGB1 | 6.291577 | 0.006991 | 2.462347 | 1.279306 | 4.739405 | worse |
| AP2M1 | 7.616178 | 0.008142 | 2.888969 | 1.316669 | 6.338831 | worse |
| SLC7A5 | 6.724629 | 0.008214 | 2.442195 | 1.259588 | 4.735131 | worse |
| CLEC11A | 3.043943 | 0.008368 | 2.160843 | 1.218707 | 3.83131 | worse |
| SERPINE1 | 6.761366 | 0.008523 | 2.176423 | 1.21928 | 3.884929 | worse |
| MYH9 | 8.359416 | 0.01073 | 2.438586 | 1.22953 | 4.836565 | worse |
| BMP2 | 1.472635 | 0.011447 | 3.400883 | 1.31695 | 8.78242 | worse |
| PLAU | 7.829211 | 0.015005 | 2.600291 | 1.203892 | 5.616375 | worse |
| COL5A1 | 6.433141 | 0.01515 | 2.335128 | 1.177854 | 4.629452 | worse |
| ITGA3 | 6.287113 | 0.016296 | 2.419932 | 1.176679 | 4.976776 | worse |
| MMP10 | 5.374764 | 0.016979 | 1.999673 | 1.132032 | 3.532314 | worse |
| LAMB3 | 7.129341 | 0.017049 | 2.240108 | 1.154786 | 4.345467 | worse |
| ACTN1 | 5.513615 | 0.017897 | 1.993424 | 1.126153 | 3.528598 | worse |
| POSTN | 6.484937 | 0.018149 | 2.069105 | 1.131908 | 3.782281 | worse |
| STIP1 | 5.780739 | 0.019085 | 1.993795 | 1.119655 | 3.550395 | worse |
| CTHRC1 | 4.784675 | 0.019591 | 1.968298 | 1.114649 | 3.475709 | worse |
| AEBP1 | 6.643334 | 0.02019 | 2.07219 | 1.120544 | 3.832043 | worse |
| FLNA | 7.446741 | 0.020382 | 1.968913 | 1.110657 | 3.490382 | worse |
| MMP11 | 5.500579 | 0.020864 | 2.056583 | 1.115582 | 3.791326 | worse |
| LGALS1 | 9.140318 | 0.022199 | 2.444177 | 1.136301 | 5.257409 | worse |
| COL16A1 | 2.840777 | 0.02328 | 0.507001 | 0.281951 | 0.911683 | better |
| IGFBP3 | 4.73384 | 0.023576 | 3.861848 | 1.198936 | 12.43925 | worse |
| ABCC5 | 4.586739 | 0.023963 | 2.013297 | 1.09668 | 3.69603 | worse |
| OAS3 | 3.680016 | 0.024141 | 2.3405 | 1.117639 | 4.901348 | worse |
| SLC3A2 | 6.345321 | 0.024315 | 1.947332 | 1.090305 | 3.47802 | worse |
| TFRC | 4.976997 | 0.025622 | 1.971361 | 1.086211 | 3.577818 | worse |
| COL4A2 | 6.814887 | 0.026519 | 2.379618 | 1.10632 | 5.118396 | worse |
| CD109 | 3.840162 | 0.026744 | 2.042038 | 1.085752 | 3.840582 | worse |
| COL12A1 | 5.405238 | 0.026963 | 1.943738 | 1.078682 | 3.502531 | worse |
| ENO2 | 2.911595 | 0.027451 | 2.468519 | 1.10565 | 5.511316 | worse |
| PHLDB2 | 2.989423 | 0.027644 | 1.9962 | 1.079031 | 3.692955 | worse |
| VCAN | 4.000157 | 0.031027 | 1.964671 | 1.06355 | 3.629291 | worse |
| PKP1 | 8.246149 | 0.031748 | 0.522561 | 0.288999 | 0.944881 | better |
| GAS2L1 | 3.997238 | 0.033976 | 0.508001 | 0.271631 | 0.950059 | better |
| COL6A3 | 5.887295 | 0.034662 | 1.933703 | 1.048702 | 3.565556 | worse |
| CA9 | 5.184535 | 0.03497 | 0.360959 | 0.140001 | 0.930645 | better |
| ASPN | 4.096514 | 0.035009 | 1.934746 | 1.047515 | 3.573451 | worse |
| FSTL3 | 3.903786 | 0.035906 | 1.880128 | 1.042409 | 3.391068 | worse |
| BGN | 8.254799 | 0.036019 | 1.929251 | 1.043792 | 3.565854 | worse |
| AMIGO2 | 1.421356 | 0.036611 | 4.530004 | 1.098611 | 18.67899 | worse |
| LRRC15 | 0.611322 | 0.036992 | 0.449613 | 0.212143 | 0.952902 | better |
| ASPM | 2.131503 | 0.038496 | 0.542379 | 0.30386 | 0.968129 | better |
| IFITM1 | 5.089614 | 0.041221 | 0.449885 | 0.208941 | 0.968676 | better |
| UBE2C | 5.795721 | 0.041342 | 0.293607 | 0.090456 | 0.953012 | better |
| FLAD1 | 3.987498 | 0.042629 | 0.507539 | 0.263466 | 0.977718 | better |
| CSF2 | 0.543284 | 0.043818 | 1.895341 | 1.017879 | 3.529222 | worse |
| OLFML2A | 4.443045 | 0.044752 | 0.348464 | 0.124463 | 0.975605 | better |
| MYO1B | 3.725389 | 0.045502 | 2.582726 | 1.01917 | 6.54501 | worse |
| PDPN | 5.344163 | 0.045782 | 1.813568 | 1.011219 | 3.252539 | worse |
| ULBP2 | 2.306184 | 0.046441 | 2.397963 | 1.013887 | 5.671466 | worse |
| SPP1 | 5.038532 | 0.046533 | 1.848931 | 1.00948 | 3.386442 | worse |
| SLC39A6 | 4.419175 | 0.046775 | 0.523781 | 0.276889 | 0.990817 | better |
| ERVMER34-1 | 0.825448 | 0.046824 | 3.334006 | 1.01706 | 10.92915 | worse |
| APOE | 6.261193 | 0.047141 | 0.514616 | 0.267061 | 0.991646 | better |
| HMGB2 | 5.762405 | 0.049198 | 0.422276 | 0.178858 | 0.996978 | better |
| COL7A1 | 5.193031 | 0.049977 | 0.546646 | 0.298839 | 0.99994 | better |
| NME1 | 4.149165 | 0.050092 | 1.769882 | 0.999772 | 3.133198 | not-significant |
| MSN | 6.86754 | 0.054996 | 1.775495 | 0.987792 | 3.191342 | not-significant |
| SPARC | 8.87513 | 0.057039 | 1.893319 | 0.981079 | 3.653791 | not-significant |
| PRC1 | 3.244303 | 0.057421 | 3.112893 | 0.964853 | 10.04308 | not-significant |
| THY1 | 4.445843 | 0.059916 | 1.757184 | 0.976738 | 3.161231 | not-significant |
| C1QTNF6 | 2.376327 | 0.060829 | 1.72819 | 0.975437 | 3.061852 | not-significant |
| DNAJC9 | 2.685257 | 0.062264 | 0.577047 | 0.323731 | 1.028579 | not-significant |
| GPC1 | 6.609642 | 0.064457 | 1.73761 | 0.967388 | 3.121072 | not-significant |
| CLIC4 | 5.810836 | 0.065148 | 1.843548 | 0.962344 | 3.531657 | not-significant |
| TNFRSF12A | 5.460711 | 0.06558 | 1.746846 | 0.964685 | 3.16318 | not-significant |
| COL1A1 | 10.21066 | 0.067079 | 1.985237 | 0.952905 | 4.135948 | not-significant |
| GLO1 | 5.475837 | 0.069373 | 2.216655 | 0.938839 | 5.233657 | not-significant |
| AGRN | 4.23726 | 0.07086 | 0.493746 | 0.229586 | 1.061844 | not-significant |
| KRT75 | 3.635135 | 0.071375 | 0.544164 | 0.280845 | 1.05437 | not-significant |
| COL6A1 | 6.216627 | 0.071385 | 0.59041 | 0.332956 | 1.046935 | not-significant |
| ODC1 | 8.941553 | 0.073456 | 0.273937 | 0.06636 | 1.130836 | not-significant |
| FZD6 | 5.336927 | 0.075459 | 1.765211 | 0.943389 | 3.302951 | not-significant |
| CCT3 | 6.241857 | 0.078768 | 1.873235 | 0.930387 | 3.771561 | not-significant |
| COL1A2 | 8.433453 | 0.07932 | 1.751462 | 0.936523 | 3.275542 | not-significant |
| COL3A1 | 10.04301 | 0.080951 | 1.91977 | 0.922874 | 3.993523 | not-significant |
| SPHK1 | 3.062315 | 0.082828 | 3.551469 | 0.84812 | 14.87164 | not-significant |
| CXCL8 | 3.762513 | 0.085591 | 1.784063 | 0.922103 | 3.45176 | not-significant |
| MCAM | 2.934929 | 0.094548 | 2.426136 | 0.85837 | 6.857336 | not-significant |
| GPRIN1 | 2.432496 | 0.096639 | 1.636966 | 0.915233 | 2.927842 | not-significant |
| FCGR2A | 3.677919 | 0.098317 | 2.075545 | 0.873219 | 4.933338 | not-significant |
| UBE2S | 4.754159 | 0.100012 | 1.985036 | 0.876885 | 4.493598 | not-significant |
| BICD2 | 5.510793 | 0.101819 | 0.554427 | 0.273517 | 1.123843 | not-significant |
| STARD4 | 2.319224 | 0.107971 | 1.598617 | 0.902211 | 2.832569 | not-significant |
| CXCL5 | 0.218767 | 0.110381 | 0.586752 | 0.304924 | 1.129061 | not-significant |
| SLC2A1 | 8.766962 | 0.110972 | 0.431344 | 0.153377 | 1.213069 | not-significant |
| PTHLH | 4.308494 | 0.116563 | 1.90902 | 0.851351 | 4.280677 | not-significant |
| ALOXE3 | 2.952098 | 0.117327 | 0.392601 | 0.121838 | 1.265089 | not-significant |
| MMP9 | 7.046263 | 0.119707 | 1.784622 | 0.86039 | 3.701665 | not-significant |
| KNSTRN | 2.20051 | 0.124187 | 4.736346 | 0.652183 | 34.39678 | not-significant |
| TGFB1 | 5.124913 | 0.125327 | 1.67046 | 0.866758 | 3.219393 | not-significant |
| ITGB4 | 7.632521 | 0.134789 | 1.716294 | 0.845548 | 3.483737 | not-significant |
| CDCA4 | 3.799856 | 0.136384 | 0.580119 | 0.283347 | 1.187726 | not-significant |
| NMB | 4.285757 | 0.137176 | 1.568536 | 0.866391 | 2.839718 | not-significant |
| JAG1 | 5.454956 | 0.143689 | 1.566652 | 0.858274 | 2.85969 | not-significant |
| YBX1 | 7.611821 | 0.14917 | 1.985448 | 0.781883 | 5.041683 | not-significant |
| PRAME | 2.265251 | 0.151 | 1.525908 | 0.85711 | 2.716566 | not-significant |
| CHI3L1 | 2.369852 | 0.155514 | 0.645466 | 0.352784 | 1.180966 | not-significant |
| EFNB1 | 5.613812 | 0.158607 | 1.583612 | 0.835763 | 3.000643 | not-significant |
| COL10A1 | 3.344008 | 0.159279 | 1.512016 | 0.850201 | 2.689002 | not-significant |
| UCN2 | 2.624313 | 0.169286 | 0.586592 | 0.274155 | 1.255093 | not-significant |
| THBS2 | 5.962863 | 0.170076 | 1.632424 | 0.810555 | 3.287635 | not-significant |
| ADRM1 | 6.374143 | 0.175567 | 0.635379 | 0.329631 | 1.224724 | not-significant |
| SLC38A2 | 5.49945 | 0.187231 | 1.870097 | 0.737618 | 4.74129 | not-significant |
| ATP13A3 | 3.195584 | 0.190079 | 1.632294 | 0.784337 | 3.396988 | not-significant |
| IL36G | 6.54972 | 0.190931 | 1.668183 | 0.774784 | 3.591756 | not-significant |
| HLA.A | 8.25451 | 0.193466 | 0.664629 | 0.359062 | 1.230239 | not-significant |
| C1orf74 | 1.708103 | 0.197682 | 1.54844 | 0.796106 | 3.011741 | not-significant |
| CSPG4 | 4.276568 | 0.204017 | 0.642144 | 0.32419 | 1.271935 | not-significant |
| ENAH | 4.157138 | 0.223223 | 0.621886 | 0.289573 | 1.335561 | not-significant |
| HDGF | 7.2723 | 0.231194 | 0.641077 | 0.309615 | 1.32739 | not-significant |
| PI15 | 0.841992 | 0.239054 | 0.709555 | 0.400792 | 1.256182 | not-significant |
| ICAM1 | 4.557635 | 0.239959 | 0.692472 | 0.37515 | 1.278202 | not-significant |
| SLC16A1 | 3.757061 | 0.243347 | 1.743515 | 0.685229 | 4.436245 | not-significant |
| ENO1 | 9.385664 | 0.250398 | 0.663454 | 0.329577 | 1.335563 | not-significant |
| HOMER3 | 3.277164 | 0.256447 | 1.971522 | 0.610449 | 6.367278 | not-significant |
| PPP1R18 | 3.767535 | 0.266081 | 0.63204 | 0.281571 | 1.418736 | not-significant |
| MIF | 5.433414 | 0.272324 | 1.444833 | 0.748974 | 2.787205 | not-significant |
| COL5A3 | 3.459039 | 0.27593 | 1.372425 | 0.776522 | 2.425623 | not-significant |
| STAT2 | 3.941907 | 0.318429 | 0.731576 | 0.395911 | 1.351829 | not-significant |
| C1orf68 | 0.672666 | 0.344499 | 0.735218 | 0.388553 | 1.391177 | not-significant |
| CTSV | 4.651434 | 0.351308 | 0.717248 | 0.356587 | 1.442692 | not-significant |
| PLAUR | 2.831581 | 0.996113 | 82169698 | 0 | Inf | not-significant |
| CEP55 | 3.034234 | 0.996386 | 77902824 | 0 | Inf | not-significant |
